# Zinc deficiency causes delayed ATP clearance and adenosine generation in rats and cell culture models

**DOI:** 10.1038/s42003-018-0118-3

**Published:** 2018-08-22

**Authors:** Taka-aki Takeda, Shiho Miyazaki, Miki Kobayashi, Katsutoshi Nishino, Tomoko Goto, Mayu Matsunaga, Minami Ooi, Hitoshi Shirakawa, Fumito Tani, Tatsuyoshi Kawamura, Michio Komai, Taiho Kambe

**Affiliations:** 1 Division of Integrated Life Science, Graduate School of Biostudies, Kyoto University, Kyoto, 606-8502 Japan; 2 Department of Science of Food Function and Health, Graduate School of Agricultural Science, Tohoku University, Sendai, 980-8572 Japan; 3 Division of Food Science and Biotechnology, Graduate School of Agriculture, Kyoto University, Kyoto, 606-8502 Japan; 4 Department of Dermatology, Faculty of Medicine, University of Yamanashi, Yamanashi, 409-3898 Japan; 5 Present Address: Faculty of Human Life Science, Miyagi Gakuin Women‘s University, Sendai, 981-8557 Japan

## Abstract

Zinc deficiency causes myriad pathophysiological symptoms, but why distinct phenotypes are generated by zinc deficiency remains unclear. Considering that several ectoenzymes involved in purinergic signaling through extracellular adenine-nucleotide hydrolysis possess zinc ions in their active sites, and disorders in purinergic signaling result in diverse diseases that are frequently similar to those caused by zinc deficiency, herein we examine whether zinc deficiency affects extracellular adenine-nucleotide metabolism. Zinc deficiency severely impairs the activities of major ectoenzymes (ENPP1, ENPP3, NT5E/CD73, and TNAP), and also strongly suppresses adenine-nucleotide hydrolysis in cell-membrane preparations or rat plasma, thereby increasing ATP and ADP levels and decreasing adenosine levels. Thus, zinc deficiency delays both extracellular ATP clearance and adenosine generation, and zinc modulates extracellular adenine-nucleotide metabolism. Since the finely tuned balance between extracellular adenine nucleotides and adenosine is critical for purinergic signaling, these findings provide a novel insight into why zinc deficiency results in diverse symptoms.

## Abstract (web summary)

Taka-aki Takeda et al. find that zinc deficiency impairs adenine nucleotide metabolism in both cell and rat models leading to delays in extracellular ATP clearance and adenosine generation. The results show that zinc deficiency affects purinergic signaling and may explain why zinc deficiency in humans results in diverse symptoms.

## Introduction

Zinc is a trace nutrient indispensable for life. It plays crucial roles in numerous biological processes, and thus, its deficiency causes myriad pathophysiological symptoms in human patients and animal models. The representative symptoms include persistent diarrhea, severe dermatitis, chronic inflammation, alopecia, taste disorders, immune insufficiency, brain dysfunction, impaired wound healing, loss of appetite, growth retardation, liver disease, and neuropsychological changes such as emotional instability, irritability, and depression (reviewed in refs. ^1–12^). The recent definition of neuromodulatory functions of zinc can explain the association between zinc and neurodegenerative diseases under zinc deficiency^13,14^. Furthermore, the regulatory roles of zinc in insulin metabolism can suggest its association with dysregulation of glucose metabolism under zinc deficiency^15,16^. However, the potential reasons for the many symptoms associated with zinc deficiency have still not been well elucidated. In contrast, iron deficiency mainly results in anemia due to the higher iron content in red blood cells. Addressing this critical question regarding zinc deficiency can potentially lead to novel therapeutic applications of zinc to improve human health. Importantly, these pathophysiological symptoms caused by zinc deficiency are often similar to those caused by dysfunctions in purinergic signaling.

**Fig. 1:**
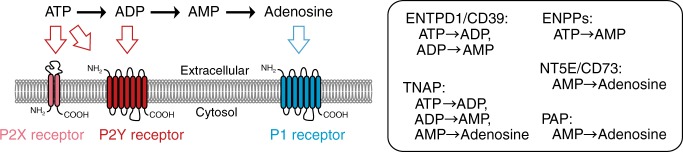
Ectoenzymes involved in extracellular adenine-nucleotide metabolism. ATP, ADP, and adenosine trigger purinergic signaling by binding to ionotropic P2X, metabotropic P2Y, and P1 receptors. ATP is hydrolyzed to ADP, AMP, and adenosine, with the hydrolysis being mediated by several ectoenzymes. P2 and P1 receptors frequently transduce signals that produce opposite effects. Representative ectoenzymes are shown in the box on the right

In purinergic signaling, extracellular adenine nucleotides and adenosine produce diverse effects in a cell-specific manner, and these effects are mediated by P2 and P1 receptors. Extracellular ATP triggers signaling events through numerous P2 (P2X and P2Y) receptors, and ADP hydrolyzed from ATP also triggers P2Y receptor signaling (Fig. 1). In contrast, adenosine, hydrolyzed from ATP through ADP and AMP, elicits a distinct signaling response through P1 adenosine receptors. Because P2 and P1 receptors frequently transduce signals that produce opposite effects, the resulting cellular response is attributable to the ratio of both ATP and ADP to adenosine and is thus involved in both physiology and pathophysiology in distinct manners^17–22^. Extracellular adenine nucleotides and adenosine are metabolized by several adenine-nucleotide-hydrolyzing ectoenzymes that mediate the hydrolysis from ATP to adenosine through ADP and AMP. Thus, the complex and integrated network of these enzymes is considered to govern the duration and magnitude of purinergic signaling. The ectoenzymes are divided into five principal groups/enzymes (Fig. 1): the ectonucleotide triphosphate diphosphohydrolase (ENTPDase) family, the ectonucleotide pyrophosphatase/phosphodiesterase (ENPP) family, the ecto-5′-nucleotidase (NT5E, also known as CD73), the alkaline phosphatase (ALP) family, and prostatic acid phosphatase (PAP)^17,18,20,22^. Specifically, the ENTPDase-family proteins play pivotal roles in the hydrolysis of extracellular ATP to ADP and ADP to AMP^23^. The ENPP proteins, particularly ENPP1 and ENPP3, are NPP-type ectophosphodiesterases, and thus are involved in the hydrolysis of extracellular ATP to AMP^24^. NT5E/CD73, the only 5′-ectonucleotidase, is regarded as the rate-limiting enzyme in the generation of extracellular adenosine through AMP dephosphorylation. The ALP-family proteins are the only ectonucleotidases that contribute to all reactions in the hydrolysis of extracellular ATP to adenosine through ADP and AMP^17^. Prostatic acid phosphatase is considered to contribute to the physiological generation of adenosine through AMP hydrolysis, although it is less characterized^25^. Considering the importance of their hydrolase activities in extracellular adenine-nucleotide metabolism, the integrated control of the functions of ectoenzymes must be operative. However, the molecular underpinnings of the activation of these enzymes remain mostly unknown.

Recent crystal structural studies revealed that the ENPP proteins and NT5E/CD73 contain two zinc ions in their active sites^26–31^, as in ALP proteins^32,33^, whereas ENTPD1/CD39 and PAP do not harbor zinc at the active sites^34,35^. This raises the possibility that zinc status is associated with extracellular adenine-nucleotide metabolism, and thus, with purinergic signaling. Herein, we test this hypothesis by directly measuring the activities of these ectoenzymes in cultured cells (both cells endogenously expressing the enzymes and cells overexpressing the enzymes) and in rats that were fed zinc-deficient diets.

## Results

### Effects of zinc deficiency on ATP/ADP/AMP-hydrolyzing enzyme

**Fig. 2:**
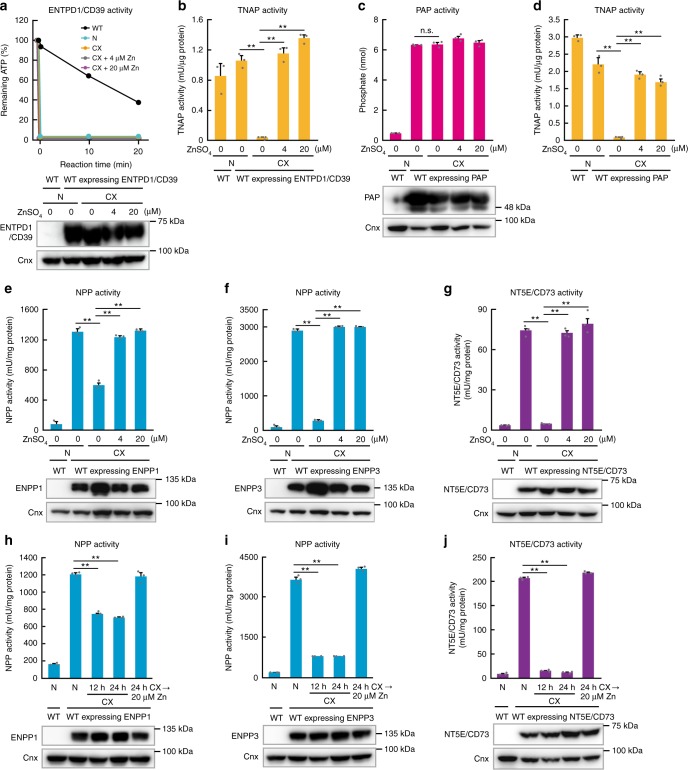
Marked reduction of ENPP1, ENPP3, and NT5E/CD73 activities in zinc-deficient cultures. **a** ENTPD1/CD39 activity in chicken DT40 cells was not impaired by zinc deficiency. ENTPD1/CD39 activity was evaluated from the relative amount of ATP remaining after ATP hydrolysis. **b** Endogenous Tnap activity was severely reduced in the cells used in **a**. **c** PAP activity in chicken DT40 cells was not impaired by zinc deficiency. **d** Endogenous Tnap activity was severely reduced in the cells used in **c**. **e** ENPP1, **f** ENPP3, and **g** NT5E/CD73 activities significantly decreased in zinc-deficient cultures. In **a**–**g**, the activity of each enzyme was measured using membrane proteins obtained from cells cultured in normal medium, CX (Chelex) medium (zinc-deficient medium), or CX medium supplemented with 4 or 20 µM ZnSO_4_ for 24 h. The zinc-deficiency-induced reduction in the activities of **h** ENPP1, **i** ENPP3, and **j** NT5E/CD73 was reversed following zinc supplementation. The cells were cultured in CX medium for 12 or 24 h, and then supplemented with 20 µM ZnSO_4_ for a final 24 h. In **a**–**j**, all activities are expressed as the mean ± SD of triplicate experiments. Representative results from three independent experiments are displayed. Expression of each enzyme was confirmed by immunoblotting (lower panels). Calnexin (Cnx) is shown as a loading control. Full-length immunoblots are shown in Supplementary Fig. 7

We evaluated the zinc dependency of several ectoenzymes by expressing the enzymes in chicken DT40 cells and comparing their activities with that of endogenous Tnap, an isozyme of ALP^36,37^. Thus, we expressed ENTPD1/CD39, PAP, ENPP1, ENPP3, and NT5E/CD73 in DT40 cells (Fig. 2a–g) and evaluated their dependence on zinc levels in the culture medium using our previous experimental strategy. The activity of endogenous Tnap substantially decreased (>95%) in zinc-deficient cultures, whereas ENTPD1/CD39 and PAP activities were almost unchanged (Fig. 2a–d), which is consistent with their recognized features; ENTPD1/CD39 contains calcium or magnesium, instead of zinc in the active site^34,38^, and PAP has been characterized as a histidine phosphatase^35^. In contrast, the activity of ENPP1, ENPP3, and NT5E/CD73 decreased in zinc-deficient cultures (Fig. 2e–g), although ENPP1 activity decreased only moderately. Notably, the diminished activity caused by zinc deficiency recovered completely following zinc supplementation (Fig. 2h–j), because Chelex-100 (CX), which was used to create zinc deficiency in fetal calf serum (FCS) (see Methods), causes deficiency of zinc and also several other trace elements, as it can chelate divalent cations.

**Fig. 3:**
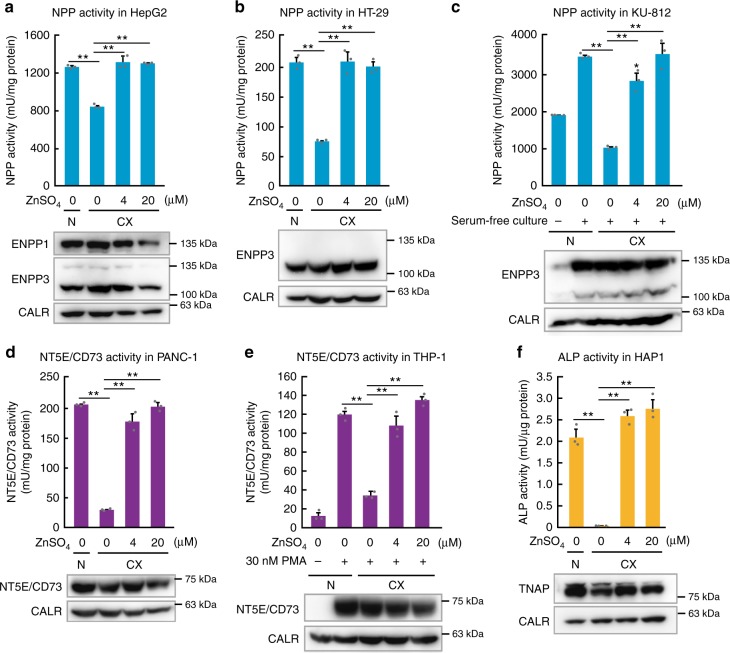
Regulation of ENPP1 or ENPP3 and NT5E/CD73 activities by zinc in human cells. Endogenous ENPP1, ENPP3, NT5E/CD73, and TNAP activities in human cells decreased in zinc-deficient cultures, and this was reversed by zinc supplementation. Enzyme activities were measured (as in Fig. 2) using **a** HepG2 cells for ENPP1 and ENPP3, **b** HT-29-MTX-E12 cells for ENPP3, **c** KU-812 cells for ENPP3, **d** PANC-1 cells for NT5E/CD73, **e** THP-1 cells for NT5E/CD73, and **f** HAP1 cells for TNAP. All activities are shown as mean ± SD of triplicate experiments. Representative results from three independent experiments are displayed. The expression of each protein was confirmed by immunoblotting (lower panels). Calreticulin (CALR) is shown as a loading control. Full-length immunoblots are shown in Supplementary Fig. 8

To complement the studies conducted using the DT40 cell overexpression system, we further examined whether the activity of ENPP1, ENPP3, and NT5E/CD73, as well as that of TNAP, is altered by zinc levels in generic human cells. Specifically, we examined ENPP1 (and ENPP3) activity in hepatoma HepG2 cells, ENPP3 activity in colon carcinoma HT-29-MTX-E12 cells and KU-812 basophils, NT5E/CD73 activity in pancreatic carcinoma PANC-1 cells and monocytic leukemia THP-1 cells, and TNAP activity in chronic myelogenous leukemia-derived HAP1 cells (Fig. 3a–f) because these are representative cells that express the corresponding ectoenzymes (Supplementary Fig. 1). KU-812 and THP-1 cells were used after differentiation because enzyme expression is enhanced following differentiation. Notably, the activity of all the measured enzymes substantially decreased in zinc-deficient cultures, which apparently caused no toxic effects on the cells (Supplementary Fig. 2), but restored by zinc supplementation in the medium (Fig. 3a–g). ENPP1 activity moderately decreased under zinc deficiency in HepG2 cells, as in ENPP1 overexpressed DT40 cells, whereas ENPP3 activity was affected significantly in HT-29-MTX-E12 and KU-812 cells. This suggests that these distinct zinc-responsive effects are due to the inherent properties of ENPP1 and ENPP3. Collectively, our findings indicate that the activity of ENPP1, ENPP3, NT5E/CD73, and TNAP, which are involved in the regulation of purinergic signaling, decreased under zinc-deficient conditions.

### Impaired ATP hydrolysis to adenosine in zinc-deficient cells

**Fig. 4:**
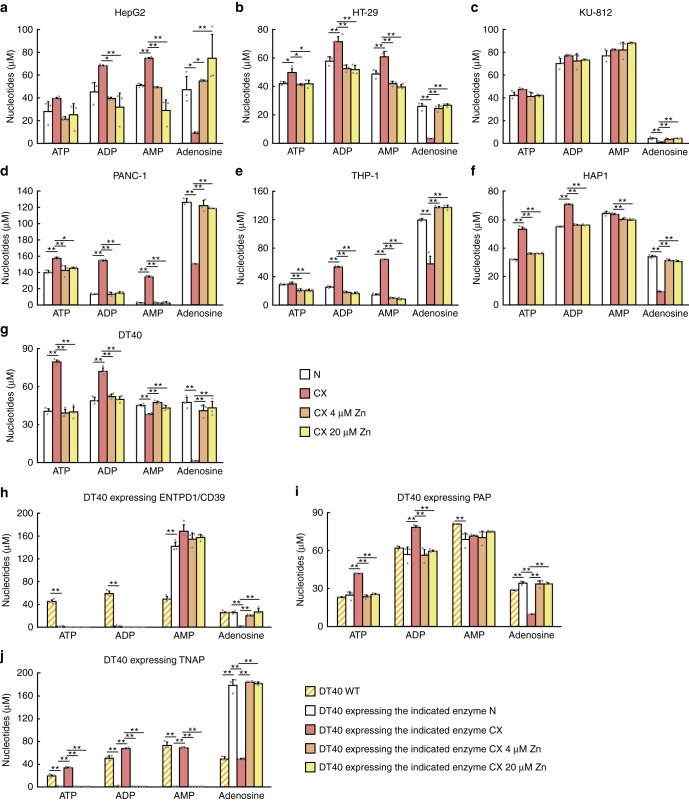
Effect of zinc status on ATP hydrolysis to adenosine. The content of ATP, ADP, AMP, and adenosine was measured after the ATP-hydrolysis reaction using membrane proteins, which were obtained from the human cell lines mentioned in Fig. 3 and from chicken DT40 cells. The cells used were **a** HepG2, **b** HT-29-MTX-E12, **c** KU-812, **d** PANC-1, **e** THP-1, **f** HAP1, **g** DT40, **h** DT40 overexpressing ENTPD1/CD39, **i** DT40 overexpressing PAP, and **j** DT40 overexpressing TNAP, which were cultured in normal medium, CX medium, or CX medium supplemented with 4 or 20 µM ZnSO_4_ for 24 h. In all cases **a**–**j**, the content of ATP, ADP, AMP, and adenosine was measured by separating them by HPLC for 20 min after hydrolysis of 200 μM ATP and then calculating the area under the corresponding peaks. The absolute amount of each molecule was calculated from the standard curves of ATP, ADP, AMP, and adenosine (Supplementary Fig. 3). Values are presented as mean ± SD of three experiments. Whereas ATP and/or ADP content increased, the adenosine content decreased in zinc-deficient cultures (CX). This was reversed by zinc supplementation (4 or 20 µM ZnSO_4_)

The results described above clearly show that the activity of each tested ectoenzyme (ENPP1, ENPP3, NT5E/CD73, or TNAP) depended on the zinc status, but the direct effects of zinc deficiency on adenine-nucleotide hydrolysis from ATP to adenosine was not clear. Thus, we addressed this point by high-performance liquid chromatography (HPLC) to directly measure the content of each adenine nucleotide and adenosine produced through ATP hydrolysis by membrane proteins obtained from the cells used in the preceding set of studies. Under the HPLC conditions used, ATP, ADP, AMP, and adenosine could be clearly discriminated (Supplementary Fig. 3a), and the peak areas corresponded to their specific amounts (Supplementary Fig. 3b). Our results showed that the amount of ATP remaining and the amount of ADP hydrolyzed from ATP were significantly higher with the use of membrane proteins obtained from all cells cultured in zinc-deficient medium (relative to control medium), whereas the adenosine content significantly decreased (Fig. 4a–f). However, their content ratio varied among the cells. These results indicate that extracellular adenine-nucleotide hydrolysis is impaired by zinc deficiency in several cell types, thus delaying both ATP clearance and adenosine generation.

A similar profile of ATP, ADP, and adenosine was found in DT40 cells, but this was significantly altered following the overexpression of ENTPD1/CD39 and TNAP, but not PAP (Fig. 4g–j). When ENTPD1/CD39 was overexpressed, the content of ATP and ADP depleted even under zinc-deficient conditions, whereas the adenosine content was almost unaltered, and these changes were accompanied by an increase in AMP content, compared with those in wild-type (WT) DT40 cells (Fig. 4g, h). In contrast, the overexpression of TNAP decreased the content of ATP, ADP, and AMP, except under zinc-deficient conditions, and thus reinforcing the effect of zinc deficiency on ATP hydrolysis (Fig. 4g, j). Collectively, our results suggest that the effects of zinc status on extracellular adenine-nucleotide metabolism are regulated by the expression pattern and level of ENTPD1/CD39, TNAP, ENPP1, ENPP3, and NT5E/CD73, with the contribution of PAP probably being negligible. Moreover, these findings indicate that zinc deficiency significantly affects adenosine generation in all the cells, likely reflecting the hydrolysis of AMP primarily by NT5E/CD73 and TNAP, which require zinc as an essential cofactor.

### Impaired ATP hydrolysis in rat plasma by zinc deficiency

**Fig. 5:**
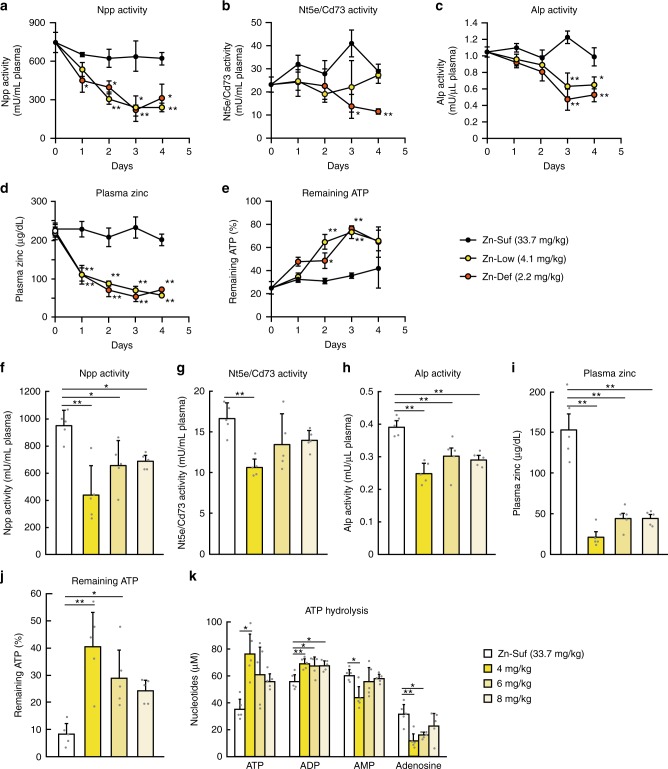
Effect of zinc deficiency on adenine-nucleotide metabolism in rat plasma. **a** Npp, **b** Nt5e/Cd73, and **c** Alp activities, and **d** zinc concentrations in rat plasma were measured. **e** ATP hydrolysis was performed using the rat plasma, and the remaining ATP was then evaluated and is expressed relative to the amount on Day 0. The plasma was obtained from the rats that were fed Zn-Def, Zn-Low, or Zn-Suf diet for up to 4 days. In **a**–**e**, values are presented as mean ± SD (*n* = 4). **f**–**j** Alp, Nt5e/Cd73, and Enpp activities, zinc concentrations, and ATP-hydrolysis rate (remaining ATP) were measured in the plasma of rats that were fed the diet containing 33.7, 4.0, 6.0, or 8.0 mg zinc per kg for 10 d. **k** The content of ATP, ADP, AMP, and adenosine after the ATP-hydrolysis reaction was measured as in Fig. 4. In **f**–**k**, values are presented as mean ± SD (*n* = 5)

To investigate how zinc deficiency affects the activity of the aforementioned ectoenzymes in vivo, we obtained plasma from rats that were fed a zinc-deficient (Zn-Def), low-zinc (Zn-Low), or zinc-sufficient (Zn-Suf) diet (2.2, 4.1, or 33.7 mg zinc per kg, respectively) (Fig. 5a–e). The ectoenzymes are all located on the cell surface, but most of them are also known to be present in the plasma. NT5E/CD73 and TNAP (and other ALPs) are released into the circulation through the hydrolysis of glycosylphosphatidylinositol anchor^29,33^, and ENPP1 and ENPP3 are converted into soluble forms through proteolytic cleavage^24^. Intriguingly, plasma Enpp (probably Enpp1 and Enpp3) activity significantly diminished one day after feeding the rats with Zn-Low and Zn-Def diets (Fig. 5a), which almost mirrored the reduction in plasma zinc concentrations in the rats of the Zn-Low and Zn-Def groups (Fig. 5d). Moreover, plasma activities of Alp (Tnap and other Alps) and Nt5e/Cd73 were significantly lower on day 3 after feeding the rats with Zn-Def and/or Zn-Low diets, compared with those in the Zn-Suf group (Fig. 5b, c). In accordance with these results, the rate of ATP hydrolysis was significantly altered in the plasma of rats in the Zn-Low and Zn-Def groups compared with that in the Zn-Suf group (Fig. 5e).

We then investigated how milder zinc deficiency (4.0, 6.0, and 8.0 mg zinc per kg) over a longer period (10 days) (relative to that in the preceding experiment) affected both ectoenzyme activities and adenine-nucleotide and adenosine content (Fig. 5f–k). The activities of Enpp, Alp, and Nt5e/Cd73 decreased in the plasma of rats that were fed tested zinc-deficient diets, with the order of activity reduction corresponding to zinc content in the diets (Fig. 5f–h). The order also agreed with the measured rate of ATP hydrolysis, which substantially changed in all the rats that were fed the zinc-deficient diets (Fig. 5j). Moreover, in the hydrolysis experiment, we examined the effect on ATP hydrolysis to adenosine in the rat plasma by quantifying the content of each adenine nucleotide and adenosine by HPLC. The results were almost identical to those obtained in the cell culture study. The amount of ATP remaining and ADP hydrolyzed from ATP markedly increased and the adenosine content significantly decreased in the plasma of rats from all groups that were fed the zinc-deficient diets (Fig. 5k).

**Fig. 6:**
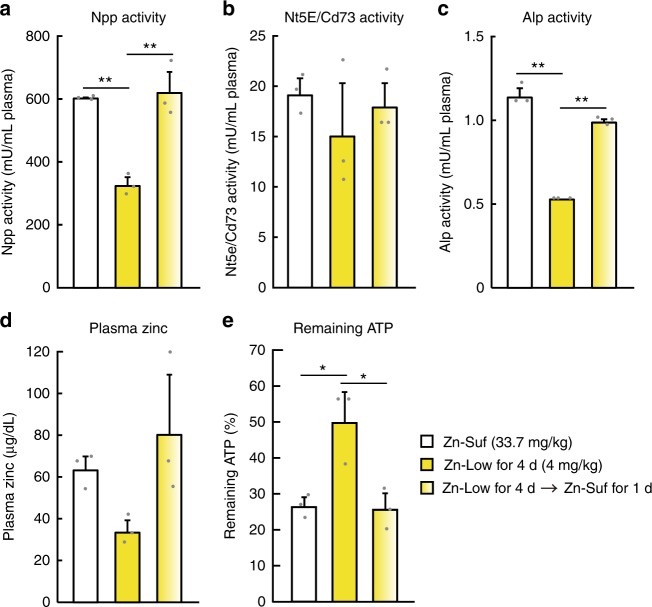
Rapid restoration of adenine-nucleotide metabolism in the plasma of rats that were fed zinc-deficient diets with 1-day zinc supplementation. **a** Npp, **b** Nt5e/Cd73, **c** Alp activities, and **d** zinc concentrations in the rat plasma were measured. **e** ATP hydrolysis was performed using the rat plasma, and the remaining ATP was then evaluated and is expressed relative to the amount on Day 0. The plasma was obtained from the rats that were fed a diet containing 4.0 mg zinc per kg for 4 days or a diet containing 33.7 mg zinc per kg for 1 day after feeding a diet containing 4.0 mg zinc per kg for 4 days. In **a**–**e**, values are presented as mean ± SD (*n* = 3)

Lastly, we examined whether the reduced enzyme activity in the plasma of rats that were fed tested zinc-deficient diets reversed by zinc-sufficient diets. The activity of Enpp, Alp, and Nt5e/Cd73 significantly decreased in the plasma four days after feeding the rats with the Zn-Low (4.0 mg zinc per kg) diet, which was reversed by the Zn-Suf (33.7 mg zinc per kg) diet for only one day (Fig. 6a–c). These results mirrored the alteration in plasma zinc concentrations (Fig. 6d), and inversely mirrored the rate of ATP hydrolysis (Fig. 6e). These results suggest that dietary zinc deficiency delays both extracellular ATP clearance and adenosine generation by reducing the activity of ectoenzymes, such as NT5E/CD73, ENPP, and ALP, and thus strongly affects the purinergic signaling cascade in vivo.

### Mechanisms underlying ENPP1, ENPP3, and NT5E/CD73 activation

**Fig. 7:**
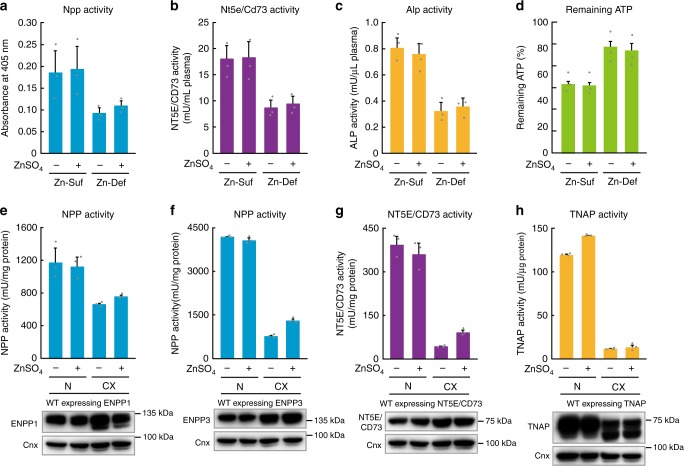
Lack of alteration of enzyme activities in both cell membrane fraction and rat plasma after incubation with zinc. **a**–**d** Rat plasma (Day 4) was incubated with 20 μM ZnSO_4_ (final concentration) at 4 °C for 24 h and then used to measure the activity of Enpp, Nt5e/Cd73, and Alp. The remaining ATP was also evaluated. **e**–**h** Membrane proteins obtained from cells expressing **e** ENPP1, **f** ENPP3, **g** NT5E/CD73, or **h** TNAP, which were cultured in normal medium or CX (Chelex) medium (zinc-deficient medium), were incubated with 20 μM ZnSO_4_ (final concentration) at 4°C for 4 h, and then used to measure enzyme activities. All activities are expressed as mean ± SD of triplicate experiments. Representative results of three independent experiments are displayed. Full-length immunoblots are shown in Supplementary Fig. 9

The reduced activity of Enpp, Nt5e/Cd73, and Alp and the rate of ATP hydrolysis in the plasma of rats that were fed the Zn-Def diet were not reversed after incubation with excess zinc (Fig. 7a–d). The same results were found with regard to the reduced activity of ENPP1, ENPP3, NT5E/CD73, and TNAP obtained from the zinc-deficient cells (Fig. 7e–h). The results indicate that these ectoenzymes are activated in the early secretory pathway^39,40^. Our previous in vitro TNAP study using DT40 cells indicated that the activation of TNAP requires two zinc-transporter complexes consisting of ZNT5–ZNT6 heterodimers and ZNT7 homodimers, which are localized in the early secretory pathway. Thus, Tnap activity was substantially decreased in chicken DT40 cells deficient in *znt5*, *znt6*, and *znt7* (triple-knockout cells; TKO cells)^41^, which suggests that the activities of ENPP1 or ENPP3 and NT5E/CD73 are regulated in a similar manner. We examined the mechanism underlying their activation.

**Fig. 8:**
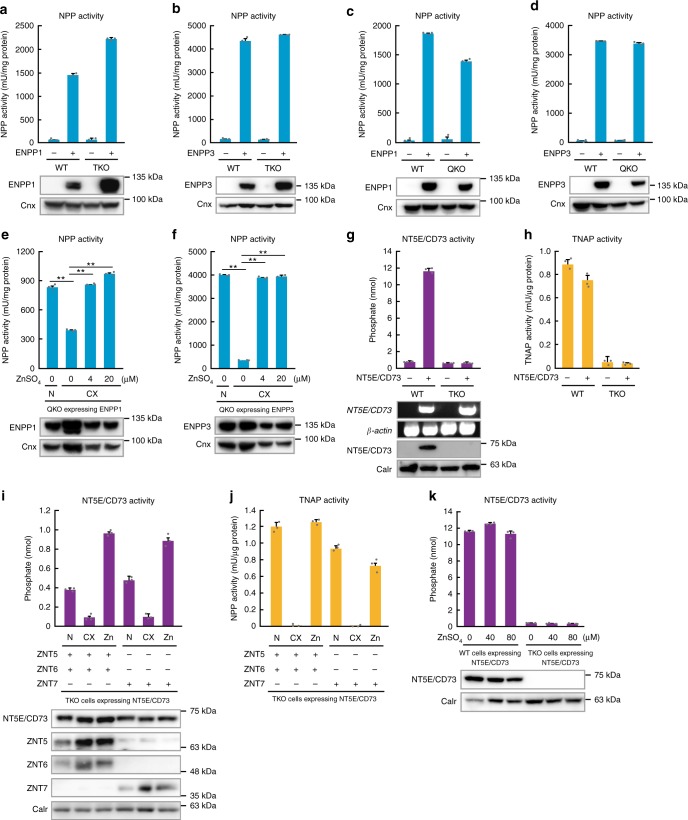
Contribution of ZNT transporters to NT5E/CD73 activity, but not ENPP1 or ENPP3 activity. **a**–**d** ENPP1 and ENPP3 activities did not decrease in either TKO or QKO cells. **e**, **f** ENPP1 and ENPP3 activities decreased in QKO cells that were cultured under zinc-deficient conditions, but this was reversed by zinc supplementation. **g** NT5E/CD73 activity significantly decreased in TKO cells. *NT5E/CD73* mRNA was expressed at almost the same level in WT and TKO cells, but NT5E/CD73 was not detected in TKO cells. **h** Endogenous Tnap activity in the cells used in **g** is shown for comparison. **i** NT5E/CD73 activity in TKO cells was restored by coexpressing ZNT5 and ZNT6 or expressing ZNT7; this restoration of NT5E/CD73 activity by ZNT5–ZNT6 heterodimers or ZNT7 homodimers required zinc in the medium. N: Normal medium; CX: zinc-deficient medium; Zn: medium supplemented with 20 µM ZnSO_4_. **j** Endogenous Tnap activity in the cells used in **i** is shown for comparison. **k** Zinc supplementation did not restore the activity of NT5E/CD73 expressed in TKO cells; the cells were cultured with 0, 40, or 80 μM ZnSO_4_ for 24 h. All enzyme activities shown in **a**–**k** were measured and are expressed as in Fig. 2. Representative results from three independent experiments are displayed. The expression of each protein was confirmed by immunoblotting (lower panels). Calreticulin (Calr) or calnexin (Cnx) is shown as a loading control. Full-length immunoblots are shown in Supplementary Figs. 10 and 11

The ENPP-family proteins feature a two-zinc-ion-centered geometry at their active site, which resembles that of ALP proteins, including TNAP^26–28,33^. This suggests that the activity of ENPP1 and ENPP3 depends on ZNT5–ZNT6 heterodimers and ZNT7 homodimers, and thus the enzymes are not activated in TKO cells, similar to that of its homolog ENPP2 (also called autotaxin)^37^, which does not significantly contribute to extracellular adenine nucleotide hydrolysis (Supplementary Fig. 4a). Unexpectedly, however, the activity of neither ENPP1 nor ENPP3 decreased in TKO cells stably expressing each of the enzymes (Fig. 8a, b). We also expressed ENPP1 and ENPP3 in quadruple-knockout (QKO) cells^37^, which lacked *znt4* in addition to *znt5*, *znt6*, and *znt7*, because the activity of another zinc-requiring ectoenzyme, carbonic anhydrase IX, decreased when expressed in QKO cells^37^. We also found that the activity of ENPP1 and ENPP3 was not altered (Fig. 8c, d), although their activities decreased in zinc-deficient cultures (Fig. 8e, f). This suggests that both ENPP1 and ENPP3 can acquire zinc through an additional pathway that is independent of all ZNT5–ZNT6 heterodimers, ZNT7 homodimers, and ZNT4 homodimers. This conclusion is in sharp contrast to that of ENPP2 because its activity almost completely decreased when expressed in TKO cells^37^. The differences between them are explained by the unique sequence features of ENPP2 because the substitution mutations of ENPP2 resulted in the gain of adenine nucleotide hydrolysis activity and ZNT transporter independency (Supplementary Fig. 4b–e).

Next, we conducted the same experiment on NT5E/CD73. In contrast to ENPP1 and ENPP3, NT5E/CD73 showed a marked reduction in activity in TKO cells, and its protein was not detected (Fig. 8g, h). Furthermore, NT5E/CD73 activity in TKO cells was restored following either coexpression of ZNT5 and ZNT6 or expression of ZNT7, although the restored NT5E/CD73 activity was lost when the cells were cultured in zinc-deficient medium (Fig. 8i, j). However, NT5E/CD73 activity in TKO cells failed to recover following the subsequent zinc supplementation in the culture medium (Fig. 8k). Thus, NT5E/CD73 is specifically activated by ZNT5–ZNT6 heterodimers or ZNT7 homodimers, as in the case of TNAP^36^. The same ZNT transporter-dependent activity was found in an ALP isozyme, placental ALP (PLAP), when expressed in TKO cells (Supplementary Fig. 5). Collectively, these findings suggest that the zinc-mediated activation of ENPP1 and ENPP3, NT5E/CD73, PLAP, and TNAP is not uniform at the molecular level, but regulated in a complex manner, and that ZNT5–ZNT6 heterodimers and ZNT7 homodimers significantly contribute to extracellular adenine-nucleotide metabolism via adenosine generation through the activation of NT5E/CD73 and TNAP.

## Discussion

To the best of our knowledge, this is the first study indicating that zinc deficiency significantly affects the activities of four ectoenzymes, ENPP1, ENPP3, NT5E/CD73, and TNAP, involved in the hydrolysis of extracellular ATP to adenosine through ADP and AMP. The detailed and focused analyses performed on ectoenzyme activity, using both in vitro cell-based studies and in vivo studies, can help elucidate the association of zinc status with purinergic signaling. Our findings were obtained using two approaches: Firstly, by directly examining the activity of each enzyme and secondly by quantifying the content of each adenine nucleotide and adenosine produced through ATP hydrolysis. In both the approaches, we used membrane proteins obtained from cells endogenously expressing or overexpressing the ectoenzymes, and we also used the plasma obtained from rats that were fed a diet containing different amounts of zinc. Notably, in this study, the cells that we used were made zinc-deficient in a physiologically relevant manner by using Chelex-100 (CX)-treated FCS, and not by using a nonphysiological equimolar amount of a potent zinc chelator such as *N*,*N*,*N*ʹ,*N*ʹ-tetrakis-(2-pyridylmethyl)-ethylenediamine, and the rats were made zinc-deficient by feeding them marginally zinc-deficient diets (such as 8.0 mg zinc per kg). Thus, the zinc status can alter extracellular adenine-nucleotide metabolism and is therefore strongly associated with the maintenance of good health and disease pathogenesis.

Limited information indicates that zinc status is associated with extracellular adenine-nucleotide metabolism, thus leading to the association with purinergic signaling. However, there is some evidence suggesting their association. Both ATP/ADP and adenosine are widely recognized to mediate opposite physiological effects, such as pain signaling by ATP and ADP vs. pain relief by adenosine, and pro-inflammatory effects of ATP vs. anti-inflammatory effects of adenosine; zinc alleviates pain^42^, whereas zinc deficiency is associated with pain^43^. Zinc is also a potent anti-inflammatory nutrient, and its deficiency leads to a pro-inflammatory state^9^. Moreover, zinc supplementation has been shown to ameliorate some defects associated with the loss of zinc-requiring ectoenzymes involved in extracellular adenine-nucleotide metabolism. Zinc masks dermatitis, whereas severe acrodermatitis is caused by decreased Langerhans cells that highly express ENTPD1/CD39 ^44,45^. Zinc can promote non-REM sleep^46^, whereas defective non-REM sleep responses to sleep deprivation are found in *Nt5e/Cd73-*KO mice^47^. Zinc can prevent diarrhea^48^, whereas allergen-induced diarrhea (inflammatory diarrhea) is found in *Enpp3*-KO mice^49^. All the diverse zinc-deficient phenotypes could not be explained by the dysfunction of extracellular adenine-nucleotide metabolism, but this information supports the results obtained in this study.

The quantification of each adenine nucleotide and adenosine hydrolyzed from ATP revealed that the effects of zinc deficiency varied depending on the cell type, which can be attributed to the distinct expression pattern and level of ENTPD1/CD39, ENPP1, ENPP3, NT5E/CD73, TNAP, PAP, and their isozymes (see Fig. 3). For example, both high expression of TNAP, ENPP1, ENPP3, and NT5E/CD73 and low expression of ENTPD1/CD39 increase the susceptibility of cells to the effects of zinc deficiency on extracellular adenine-nucleotide metabolism. In particular, our results strongly suggest that adenosine generation is readily affected by zinc deficiency. This could be why zinc deficiency symptoms do not appear uniformly in the body. Moreover, the expression pattern of ZNT5–ZNT6 heterodimers and ZNT7 homodimers may also contribute to the susceptibility to zinc deficiency, because both zinc-transporter complexes are indispensable for the activity of TNAP and NT5E/CD73 (see Fig. 8).

Another intriguing aspect that was revealed in this study is that zinc-requiring ectoenzymes show distinct ZNT transporter dependencies for their activation and furthermore exhibit differences in their susceptibility to zinc deficiency. For example, both ENPP1 and ENPP3 lost their activity under zinc-deficient conditions, with ENPP3 activity being affected more drastically (see Figs. 2e, f and 3a–c). Moreover, ENPP1 and ENPP3 did not lose their activity even when expressed in TKO and QKO cells (see Fig. 8a–d). This ZNT transporter independency of ENPP1 and ENPP3 is particularly interesting because the activity of their homolog ENPP2 is completely dependent on ZNT transporters^37^. However, the differences can be explained by the unique sequence of ENPP2 (Supplementary Fig. 4b–e). In contrast, NT5E/CD73 is completely dependent on ZNT transporters for both its activity and stability, as in the case of TNAP^36^ and PLAP (Supplementary Fig. 5). These results provide an interesting and important hypothesis that the ZNT transporter-dependent activation of each ectoenzyme is not defined by the manner of zinc coordination at the active site of zinc-requiring ectoenzymes, but rather by the functional role in physiology. Further investigation is required to clarify this, which provides a new direction for the therapeutic potential of zinc.

In conclusion, our findings have revealed that zinc deficiency potently decreases the activities of extracellular adenine-nucleotide-hydrolyzing ectoenzymes, delaying both extracellular ATP clearance and adenosine generation. These ectoenzymes are activated by zinc in the early secretory pathway, but the activation mechanism is not uniform. Our current results provide novel insight into why zinc deficiency produces diverse symptoms, considering that defects in purinergic signaling frequently result in similar phenotypes, although not all zinc-deficiency-related phenotypes can be explained by the effect on nucleotide metabolism. The results partly justify why attention should be paid to zinc deficiency to achieve good health.

## Methods

### Cell culture

Chicken B lymphocyte-derived DT40 cells were maintained in RPMI1640 (Nacalai Tesque, Kyoto, Japan) supplemented with 10% heat-inactivated FCS (Multiser, Trace Scientific, Melbourne, Australia), 1% chicken serum (Life Technologies, Grand Island, NY, USA), and 10 μM 2-mercaptoethanol (Sigma, St. Louis, MO) at 39.5 °C. HT-29-MTX-E12 cells (DS Pharma Biomedical, Co., Ltd, Osaka, Japan) and HepG2 cells were maintained at 37 °C in a humidified 5% CO_2_ incubator; the culture medium used was Dulbecco's Modified Eagle's medium (DMEM) (Sigma) containing 10% heat-inactivated FCS, 100 U/mL penicillin, and 100 μL/mL streptomycin. Rather than DMEM, RPMI1640 medium was used to maintain KU-812 cells (JCRB Cell Bank, Osaka, Japan), PANC-1 cells (provided by Dr. Shuichi Enomoto, Okayama University, Japan), and THP-1 cells (JCRB Cell Bank), and IMDM was used for HAP1 cells (Horizon, Cambridge, UK). KU-812 and THP-1 cells were used after differentiation into basophilic or macrophage-like cells by means of serum-free culture for 72 h or treatment for 48 h with 30 nM (18.5 ng/mL) phorbol 12-myristate 13-acetate (PMA; Wako Pure Chemicals, Osaka, Japan), respectively. To generate zinc-deficient culture medium, FCS or chicken serum was treated with Chelex-100 resin for 24 h, and the resin was removed by filtration. For zinc-supplementation experiments, the cell culture medium containing the indicated concentrations of ZnSO_4_ was added. In the Alamar Blue assay, the cells were inoculated at a density of 1 × 10^4^ cells per mL in 96-well plates and treated with ZnSO_4_ at the indicated concentrations for one day, and then Alamar Blue reagent (AbD Serotec, Ltd, Oxford, UK) was added to the culture medium and incubated for 4 h. Relative proliferation rate was calculated based on the absorbance at 570 and 600 nm in the medium, according to the protocol of the manufacturer.

### Plasmid construction and transfection into DT40 cells

The fragment containing human *NT5E/CD73* cDNA or *PAP* cDNA used in this study was amplified through the RT-PCR performed using the human skeletal muscle cDNA (DV Biologics, Costa Mesa, CA) or human pancreatic islet cDNA (Cosmo Bio Co. Ltd, Tokyo, Japan) as the template, and inserted into the pA-puro or pA-Ecogpt vector. The amplified cDNA was sequenced in both directions. The Human *ENPP3* cDNA was purchased from DNAFORM (Tokyo, Japan) and inserted into the pA-puro or pA-Ecogpt vector. The human *ENTPD1/CD39* cDNA was purchased from GE Healthcare Dharmacon (Buckinghamshire, UK). The plasmids used for the expression of human TNAP, FLAG-tagged human ZNT5 (FLAG-ZNT5), Halo-ZNT5, HA-ZNT6, or ZNT7-FLAG was constructed by two-step PCR methods^36,50^. All plasmids were linearized with appropriate restriction enzymes. To transfect the DNA into DT40 cells through electroporation, 1 × 10^7^ cells were suspended in 0.5 mL of cold PBS containing 30 μg of linearized plasmid DNA. DNA was electroporated into the cells with a GenePulser Xcell apparatus (Bio-Rad, Hercules, CA, USA) at 550 V and 25 μF. After electroporation, cells were transferred into 30 mL^−^^1^ of fresh medium and incubated for 24 h. Then, cells were resuspended in 60 mL of fresh medium containing appropriate drugs and divided into four 96-well plates for selection. More than three independent stable clones were established per transfectant in all experiments.

### Preparation of membrane proteins

The membrane proteins were obtained from the cells lysed either in ALP lysis buffer for exclusively measuring TNAP activity, or in NT5E/CD73 lysis buffer (10 mM Tris-HCl, pH 7.5, 0.5 mM MgCl_2_, 0.1% NP-40) to measure ENTPD1/CD39, PAP, NT5E/CD73, ENPP, or TNAP activity.

### Measurement of ALP activity

Three micrograms of membrane protein lysed in ALP lysis buffer or 5 μL of rat plasma were preincubated for 10 min at room temperature. A 100 μL substrate solution [2 mg/mL disodium *p*-nitrophenylphosphate hexahydrate (*p*NPP; Wako Pure Chemicals) in 1 M diethanolamine buffer, pH 9.8, containing 0.5 mM MgCl_2_] was added. After incubation for 10 min at room temperature, the released *p*-nitrophenol was quantified by measuring the absorbance at 405 nm. Calf intestine alkaline phosphatase (Promega, Madison, WI) was used to generate a standard curve. To measure PLAP activity, the samples were incubated at 65°C for 30 min to discriminate other activity (PLAP is heat-stable). *p*NPP is not hydrolyzed by ENPP or NT5E/CD73 (Supplementary Fig. 6a).

### Measurement of ENPP activity

ENPP activity was measured as described elsewhere^51^ with certain modifications. Briefly, 3 μg of membrane proteins or 5 μL of rat plasma was incubated at 37 °C for 10 min, and then 100 μL of 0.1 mM *p*-nitrophenyl thymidine 5′-monophosphate (*p*NP-TMP; Sigma) solution was added and incubated at 37 °C for 15 min. *p*-Nitrophenol released from *p*NP-TMP by ENPPs was quantified by measuring the absorbance at 405 nm. Human recombinant ENPP1 (R&D Systems, Minneapolis, MN) was used to generate a standard curve. *p*NP-TMP is not hydrolyzed by ENTPD1/CD39, NT5E/CD73, or TNAP (Supplementary Fig. 6b, f). Specificity of substrates for measuring enzyme activity is shown in Supplementary Fig. 6.

### Measurement of NT5E/CD73 and PAP activity

NT5E/CD73 activity was measured using a 5′-nucleotidase assay kit (Diazyme Laboratories, Poway, CA) with 3 μg of membrane proteins or 5 μL of rat plasma. Bovine NT5E/CD73 obtained from serum (Diazyme Laboratories) was used to generate a standard curve. The activity of PAP and NT5E/CD73, in certain experiments, was evaluated by the malachite green assay as described elsewhere^52^, with a few modifications. Briefly, we incubated 5 μg of membrane proteins in 200 μL of NT5E/CD73 lysis buffer at 37 °C for 10 min, and the solution was then incubated with 2 mM AMP (Oriental Yeast Co., Ltd, Tokyo, Japan) at 37 °C for 30 min. Subsequently, the amount of inorganic phosphate released from AMP was quantified by measuring the absorbance at 595 nm. For the measurement, 50 μL of the reaction solution was mixed with 100 μL of Biomol Green Reagent (Enzo Life Sciences, Plymouth Meeting, PA) and incubated for 10 min at room temperature. The specificity of NT5E/CD73 activity measured here was confirmed using α,β-methylene-ADP (Sigma), a selective inhibitor of NT5E/CD73 (Supplementary Fig. 6e). The standard curve was generated using a solution of Na_2_HPO_4_ dissolved in NT5E/CD73 lysis buffer. Specificity of substrates for measuring enzyme activity is shown in Supplementary Fig. 6.

### Measurement of ENTPD1/CD39 (ATP-hydrolysis) activity

The hydrolysis of ATP was evaluated by the firefly luciferase-based method using the ATP assay reagent (Toyo Beanet, Tokyo, Japan). We incubated 10 µg of membrane proteins or 10 μL of plasma in 900 μL of HBSS buffer containing 20 mM Hepes at 37 °C for 10 min, and then 100 μL of 1 μM ATP solution was added and the mixture was incubated at 37 °C for 40 min, and finally the reaction mixture was placed on ice. For the ATP assay, 50 μL of the reaction solution was used. Firefly luciferase activity was measured after mixing the mixture by pipetting for 10 s. The amount of ATP remaining was calculated from the luciferase activity and expressed relative to that at 0 min. ENTPD1/CD39 does not hydrolyze *p*NP-TMP (Supplementary Fig. 6f).

### Immunoblotting analysis

Membrane proteins (20 μg) were mixed with 6× sodium dodecyl sulfate (SDS) sample buffer and then incubated at 37 °C for 10 min before electrophoresis. Proteins were separated by electrophoresis using 6% or 8% SDS-polyacrylamide gels. After transfer of proteins to PVDF membranes (Millipore, Bedford, MA), the blots were blocked with PBS containing 4% skimmed milk and 0.1% Tween-20, and then incubated with the following antibodies diluted in the blocking solution: anti-HA HA-11 (1:3000; catalog no. MMS-101P; Biolegend, San Diego, CA), anti-FLAG M2 (1:3000; catalog no. F3165; Sigma), anti-NT5E/CD73 (1:3000; catalog no. 13160; Cell Signaling Technology, Beverly, MA), anti-ENPP1 (1:3000; catalog no. NBP2-27561; Novus Biologicals, Littleton, CO), anti-ENPP3 (1:3000; catalog no. HPA043772; Sigma), anti-ENTPD1/CD39 (1:1000; catalog no. sc-33558; Santa Cruz Biotechnology, Santa Cruz, CA), anti-PAP (1:3000; catalog no. P5664; Sigma), anti-calnexin (1:6000; catalog no. ADI-SPA-860; Enzo Life Sciences), anti-calreticulin (1:5000; catalog no. PA3-900; Affinity Bioreagents, Golden, CO), anti-TNAP (1:2000; catalog no. sc-30203; Santa Cruz Biotechnology), anti-ZNT5 (1:2000)^36^, or anti-placenta alkaline phosphatase L-19 (1:3000; catalog no. sc-15065; Santa Cruz Biotechnology). Immunoreactive bands were detected with horseradish peroxidase-conjugated anti-mouse or anti-rabbit secondary antibodies (1:3000; NA931 or NA934; GE Healthcare, Milwaukee, WI), or anti-goat secondary antibody (catalog no. sc-2020; Santa Cruz Biotechnology), and fluoro-images were obtained using ImageQuant LAS500 (GE Healthcare).

### RT-PCR

The total RNA was isolated from harvested DT40 cells using Sepasol I (Nacalai Tesque) and reverse-transcribed using ReverTra Ace (TOYOBO, Osaka, Japan). The PCR was performed using KOD-FX (TOYOBO). The primer sequences used in this study are shown in Supplementary Table 1.

### HPLC analysis

Either 10 μg of membrane proteins in NT5E/CD73 lysis buffer or 25 μL of plasma was incubated with ATP (at a final concentration of 200 μM in lysates prepared using 100 mM Tris-HCl, 0.5 mM CaCl_2_, 0.5 mM MgCl_2_, pH 7.5) at 37°C for 2 h (for DT40 cells and plasma) or 4 h (for human cell lines), and the samples were then stored on ice. To separate ATP, ADP, and AMP as single peaks, analyses were performed using an Inertsil ODS-SP column (5 µm, 4.6 mm × 250 mm; GL Sciences Inc., Tokyo, Japan), with a linear gradient (65% buffer containing 20 mM NH_4_H_2_PO_4_ and 12.5 mM tetra-*n*-butylammonium hydroxide plus 35% methanol) at 35 °C for 20 min, on a Chromaster HPLC system (Hitachi High-Technologies Corp., Tokyo, Japan). The flow rate was maintained at 0.7 mL/min, and sample elution was monitored at 260 and 220 nm (with 220 nm being used to trace possible contaminants). A commercially available adenosine kit (Jena BioScience GmbH, Jena, Germany) was used to prepare the standard curve, and the content of ATP, ADP, AMP, and adenosine was calculated from the standard curve based on the HPLC peak area of the absorbance at 260 nm.

### Animal care and dietary zinc manipulation

Four-week-old male Sprague-Dawley rats were purchased from Japan SLC (Hamamatsu, Japan), and acclimated for 3 days on the Zn-Suf diet. After acclimation, the rats were divided into three groups and fed one of these diets for 4 days: Zn-Def, Zn-Low, and Zn-Suf diets, which contained 2.2, 4.1, and 33.7 mg zinc per kg, respectively^53^ (Experiment 1). In another experiment, the rats were divided into four groups, after which they were allowed to acclimate for 4 days and fed one of these diets for 10 days: 4.0, 6.0, 8.0, and 33.7 mg zinc per kg diets (Experiment 2). Alternatively, the rats were divided into two groups, after which they were allowed to acclimate for 3 days, and fed a diet containing 4.0 mg zinc per kg for 4 days, or a diet containing 33.7 mg zinc per kg for 1 day after feeding the diet containing 4.0 mg zinc per kg for 4 days (Experiment 3). The rats were housed in individual stainless-steel cages under controlled conditions (temperature, 23 ± 2 °C; humidity, 50 ± 10%; 12:12-h light–dark cycle, 08:00 AM to 08:00 PM), and were sacrificed through decapitation after being food-deprived for 5 h. Blood samples were collected at the time points specified in each experiment and used to measure enzyme activity and zinc concentration in the plasma. For zinc-supplementation experiments using rat plasma, the plasma was incubated with 20 μM ZnSO_4_ (final concentration) for 24 h and then used to measure enzyme activities. The experimental protocol for this study was approved by the Animal Research-Animal Care Committee of Tohoku University and Kyoto University, and all animal experimental procedures were implemented in accordance with the guidelines issued by this committee and the Japanese governmental legislation (2005). The same committee also supervised the care and use of the rats used in this study.

### Statistical analyses

All data are presented as mean ± SD. Statistical significance was assessed by the one-way analysis of variance followed by Tukey’s test and accepted at *P* < 0.05 (*) or *P* < 0.01 (**).

### Data availability

All data generated or analyzed during this study are included in this published article (and its supplementary information files) or are available from the authors upon reasonable request. Full-length immunoblots corresponding to images in the main and supplementary figures are shown in Supplementary Figs. 7–15.

## Electronic supplementary material

Supplementary Information
