# Supplementary material for: Zinc deficiency causes delayed ATP clearance and adenosine generation in rats and cell culture models
Source: Commun Biol. 2018 Aug 22;1:113. doi: 10.1038/s42003-018-0118-3 (PMC6123718; doi:10.1038/s42003-018-0118-3)
Supplement: Supplementary file 1 — Supplementary Information [file 42003_2018_118_MOESM1_ESM.pdf]

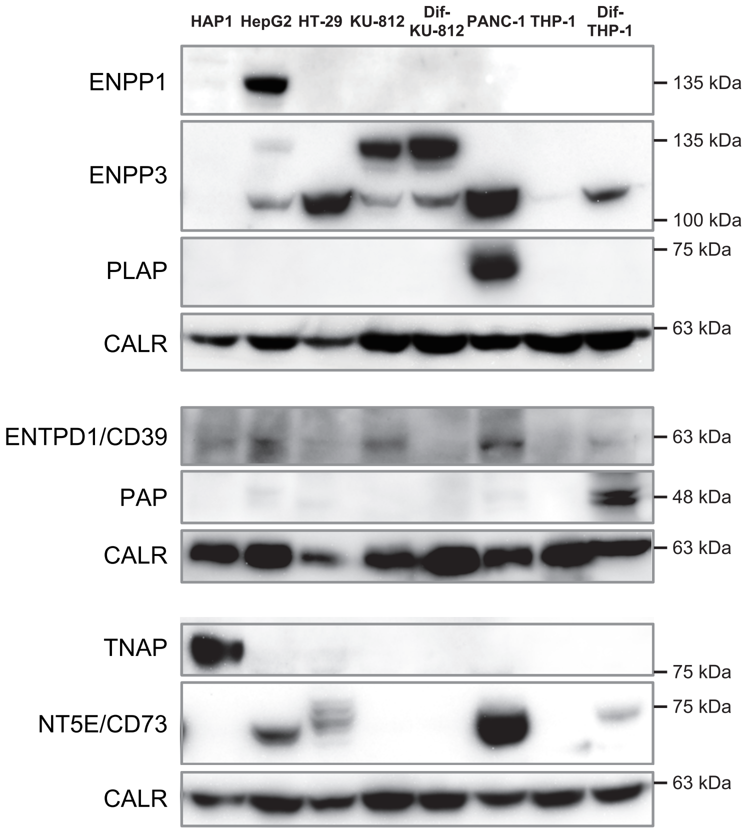

Supplementary Figure 1. Expression of zinc-requiring ectoenzymes in human cells. Immunoblotting was performed to examine the expression level of ENPP1, ENPP3, placenta alkaline phosphatase (PLAP), PAP, TNAP, ENTPD1/CD39, and NT5E/CD73 in HAP1, HepG2, HT-29-MTX-E12, KU-812, differentiated KU-812 (Dif-KU-812), PANC-1, THP-1, and differentiated THP-1 (Dif-THP-1) cells. The expression of an isozyme of ALP, PLAP, was also examined. Calreticulin (CALR) is shown as a loading control.

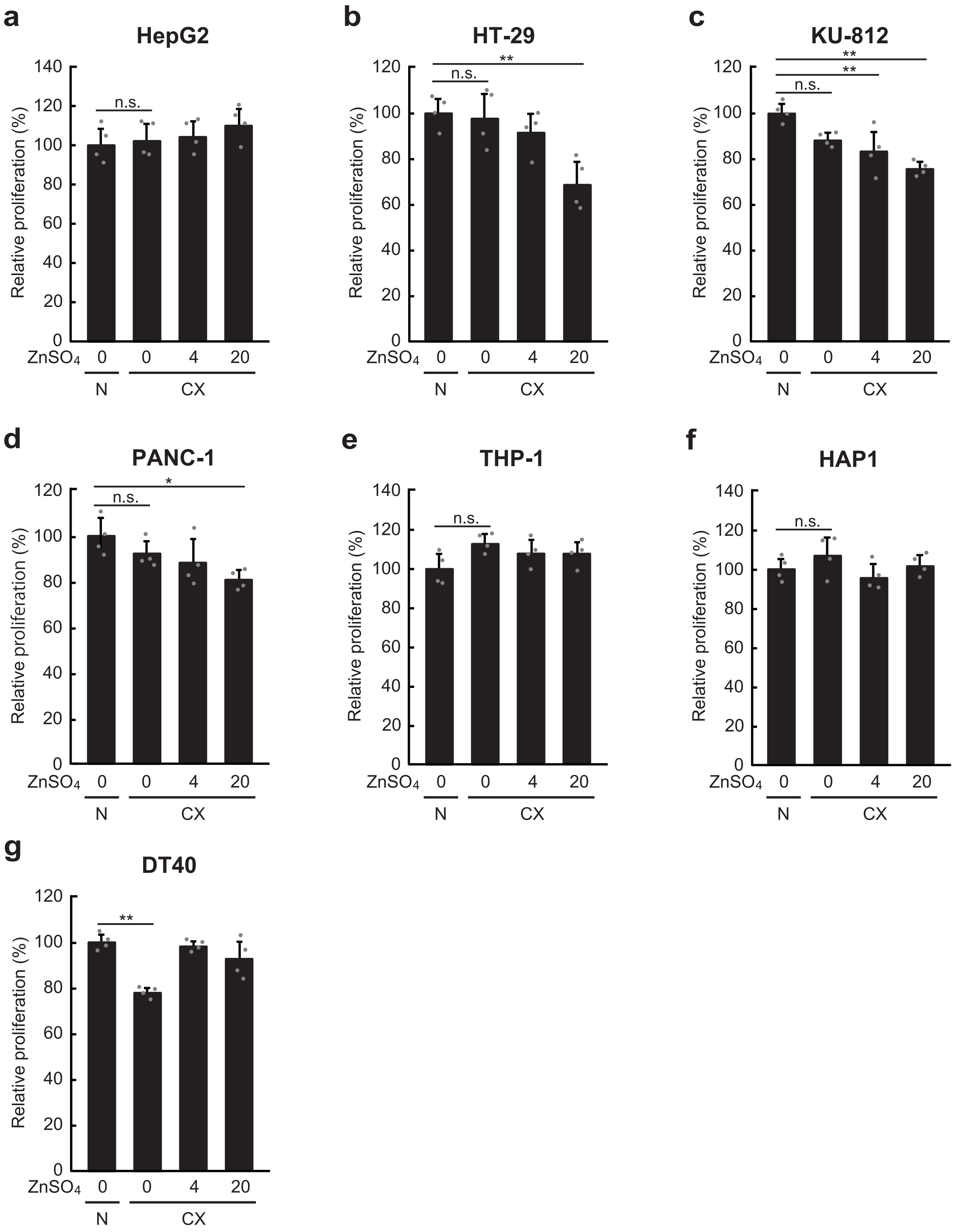

Supplementary Figure 2. Effects of zinc-deficient culture medium on the cells. Human cell lines mentioned in Fig. 3 were maintained in zinc-deficient culture media, or in those reversed by zinc supplementation for 24 h, and their number was evaluated by the Alamar Blue assay. The cells evaluated are (**a**) HepG2 cells, (**b**)

HT-29-MTX-E12 cells, (**c**) KU-812 cells, (**d**) PANC-1 cells, (**e**) THP-1 cells, and (**f**) HAP1 cells. (**g**) The result of DT40 cells is also shown. Relative proliferation is presented as mean ± SD of quadruplicate experiments. Representative results from four independent experiments are displayed.

a

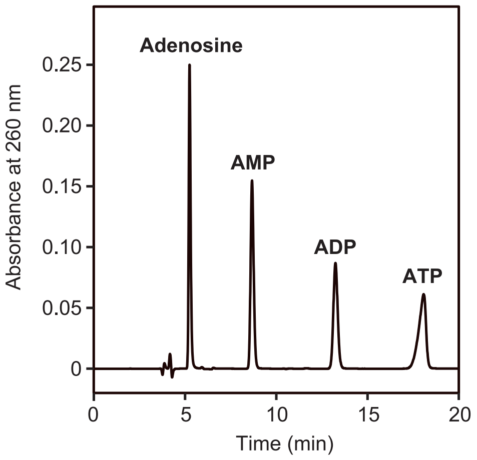

b

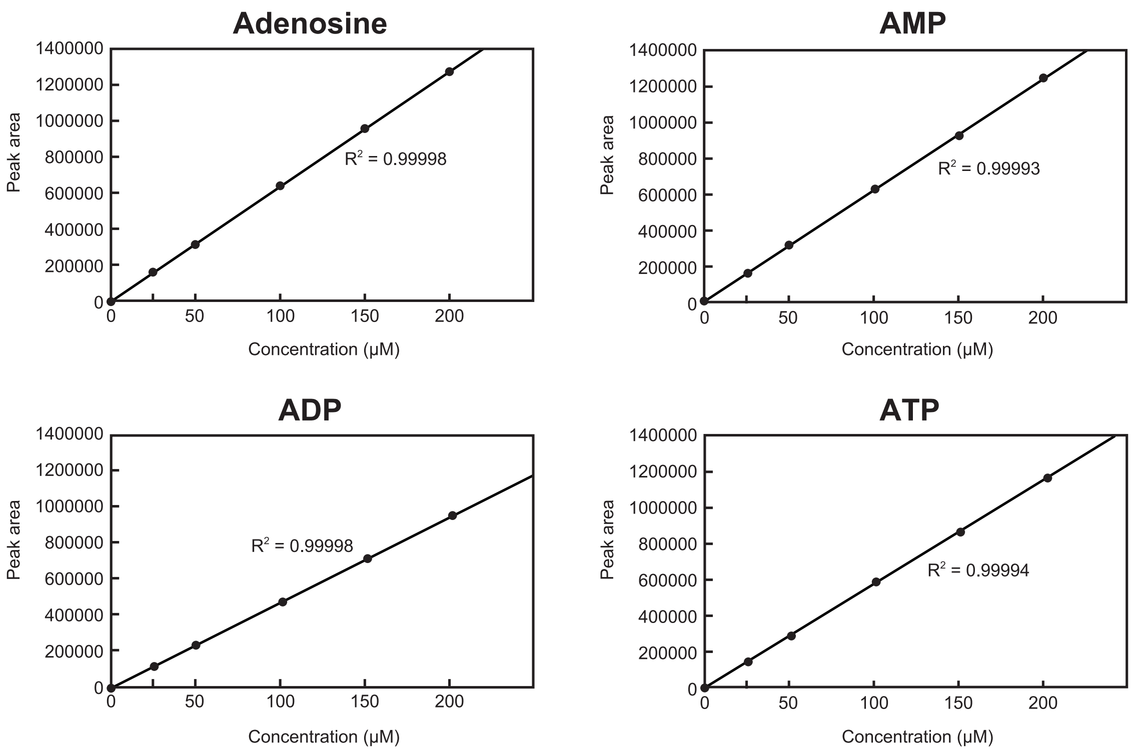

Supplementary Figure 3. Confirmation of accurate quantification by HPLC. (**a**) Representative HPLC chromatograms for ATP, ADP, AMP, and adenosine. The concentration of all chemicals was 150 μM. (**b**) Confirmation that the peak area corresponds to the amount of each adenine nucleotide and adenosine in the HPLC

analysis. The relationship between concentration plots of adenine nucleotides or/and adenosine and peak area was estimated by linear regression analysis using the least-squares method.

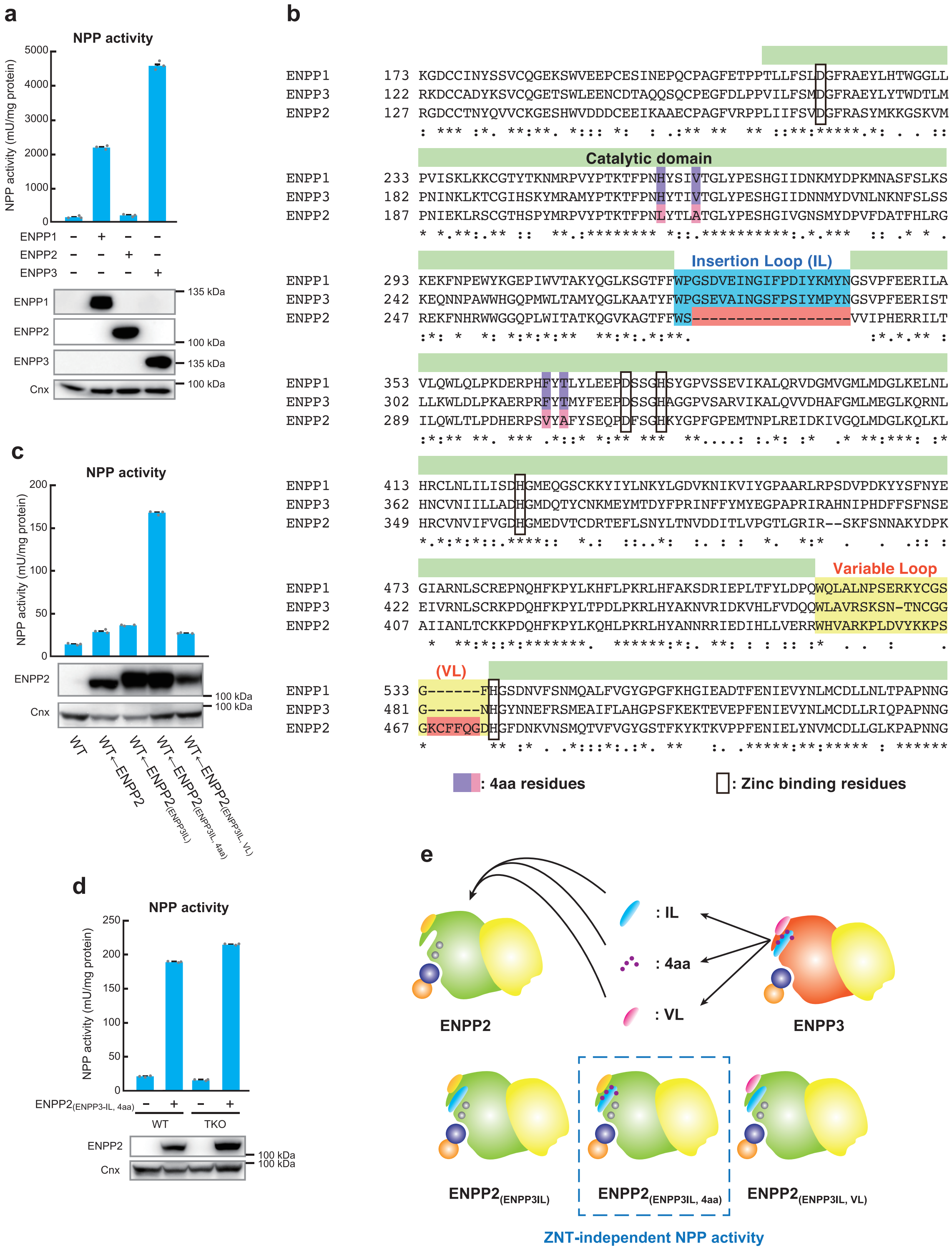

Supplementary Figure 4. NPP activity and ZNT transporter-dependency of ENPP2. ENPP2 has lysophospholipase D activity, which is activated by zinc in a ZNT transporter-dependent manner, as previously described [1], but its NPP activity has not

been examined previously. (**a**) ENPP2 exhibited almost no NPP activity, compared with ENPP1 and ENPP3. (**b**) Alignment of ENPP1, ENPP2, and ENPP3. Note that ENPP2 lacks the insertion loop (IL), and has a variable loop (VL) and substituted amino acids (4aa) in its catalytic domain, as described previously [2]. (**c**) The ENPP2 mutant, $ENPP2_{(ENPP3IL,\ 4aa)}$, in which IL of ENPP3 was inserted and 4aa of ENPP2 was substituted with those of ENPP3, can restore NPP activity. Note that both IL insertion and 4aa substitution are required for ENPP2 to acquire NPP activity. The VL is not involved in this process. (**d**) The NPP activity of $ENPP2_{(ENPP3IL,\ 4aa)}$ did not decrease in TKO cells when expressed stably. (**e**) Schematic representation of the findings in (a)-(d). All activities are presented as mean ± SD of triplicate experiments. Representative results of three independent experiments are displayed.

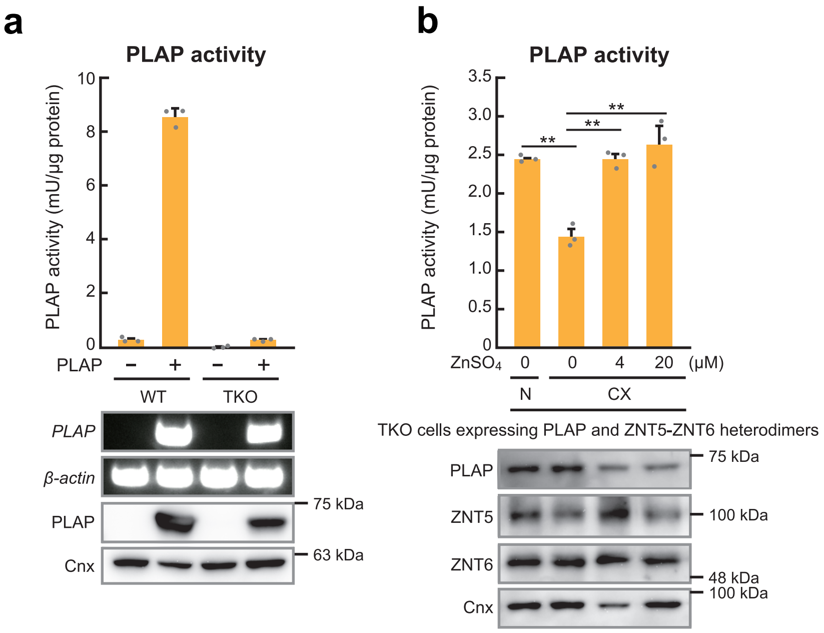

Supplementary Figure 5. ZNT transporter-dependent and Zinc-dependent PLAP activation. (**a**) PLAP activity significantly decreased in TKO cells stably expressing PLAP. (**b**) The decreased PLAP expression was restored by the co-expression of Halo-ZNT5 and HA-ZNT6. All activities are presented as mean ± SD of triplicate experiments. Representative results of three independent experiments are displayed. The expression of each enzyme was confirmed by immunoblotting (*lower* panels). Calnexin (Cnx) is shown as a loading control.

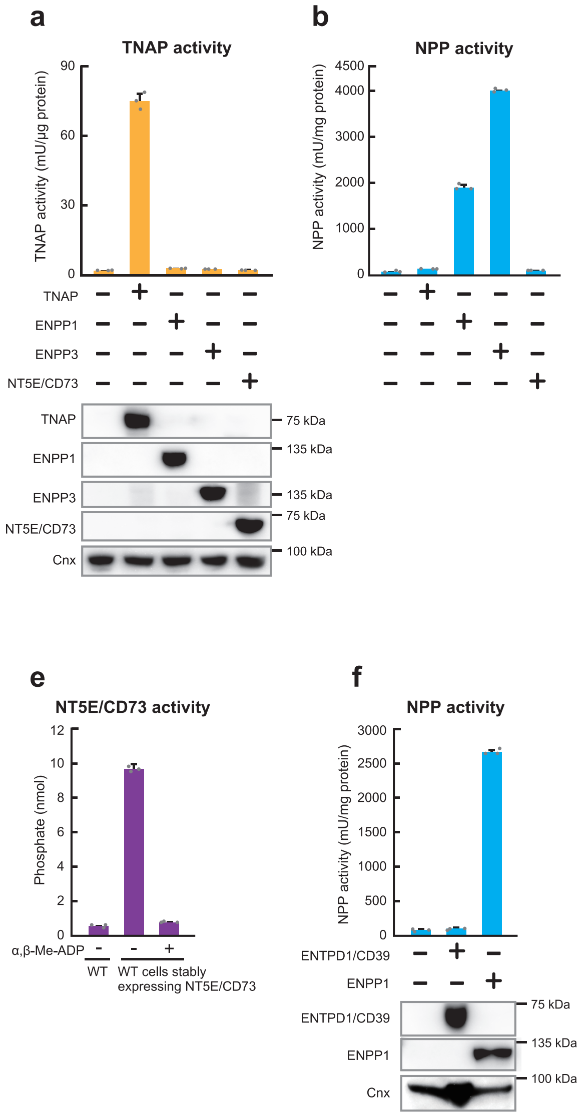

Supplementary Figure 6. Specificity of substrates to measure enzyme activity. The assays were performed using membrane proteins obtained from WT DT40 cells overexpressing TNAP, ENPP1, ENPP3, or NT5E/CD73. (**a**) TNAP activity was specifically measured using *p*NPP as a substrate. (**b**) ENPP1 and ENPP3 activities were specifically measured using *p*NP-TMP. (**c, d**) NT5E/CD73 activity was measured using the 5′-nucleotidase assay kit (c) or by the malachite green assay (d). TNAP activity was determined by these methods, and it can be discriminated by comparing with the hydrolyzing activity of *p*NPP, as shown in (a). (**e**) NT5E/CD73 activity can also be

confirmed using α,β-methylene-ADP, a selective inhibitor of NT5E/CD73. In this assay, α,β-methylene-ADP (final concentration, 200 μM) was incubated at 37°C for 30 min with the samples before incubation with a substrate. (**f**) ENTPD1/CD39 failed to hydrolyze *p*NP-TMP. In (a-f), all activities are presented as mean ± SD of triplicate experiments. Representative results of three independent experiments are displayed. The expression of each enzyme was confirmed by immunoblotting (*lower* panels). Calnexin (Cnx) is shown as a loading control.

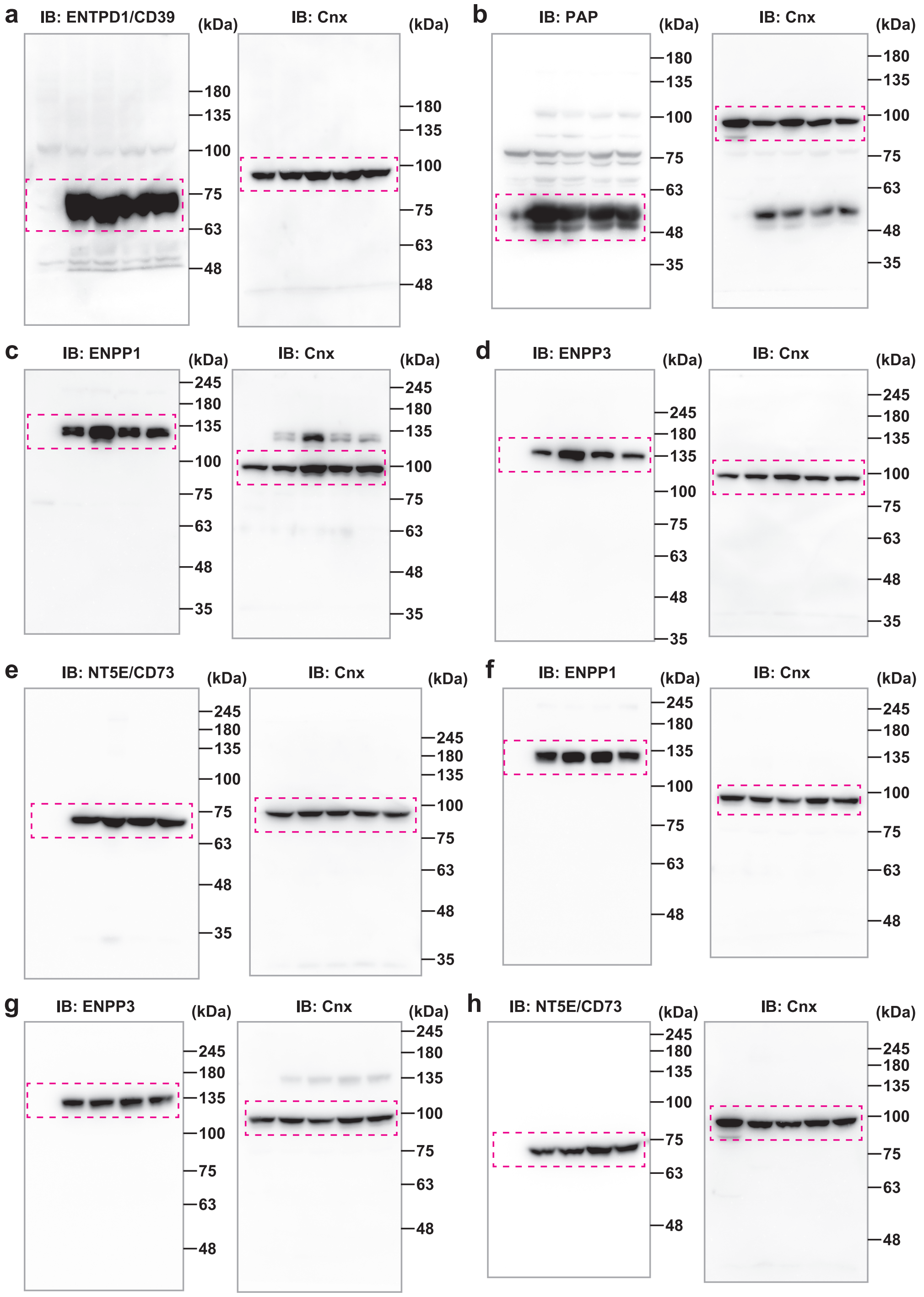

Supplementary Figure 7. Full-length immunoblot images used in Figure 2. The panel used is boxed. The same blot was used sequentially (after stripping) for detection in each composite figure. The molecular weights of the marker proteins are indicated on the right of the immunoblot images.

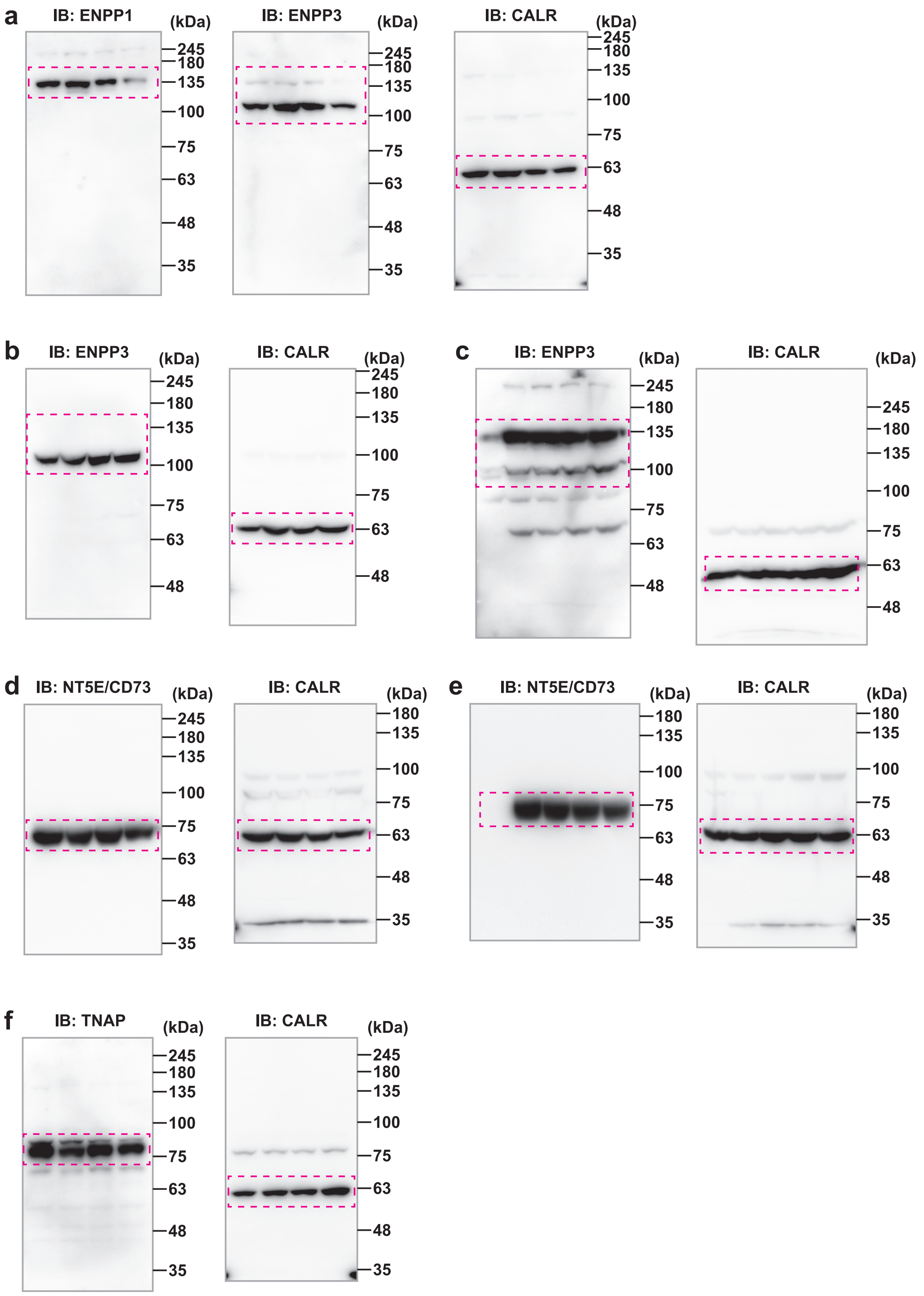

Supplementary Figure 8. Full-length immunoblot images used in Figure 3. The panel used is boxed. The same blot was used sequentially (after stripping) for detection in each composite figure. The molecular weights of the marker proteins are indicated on the right of the immunoblot images.

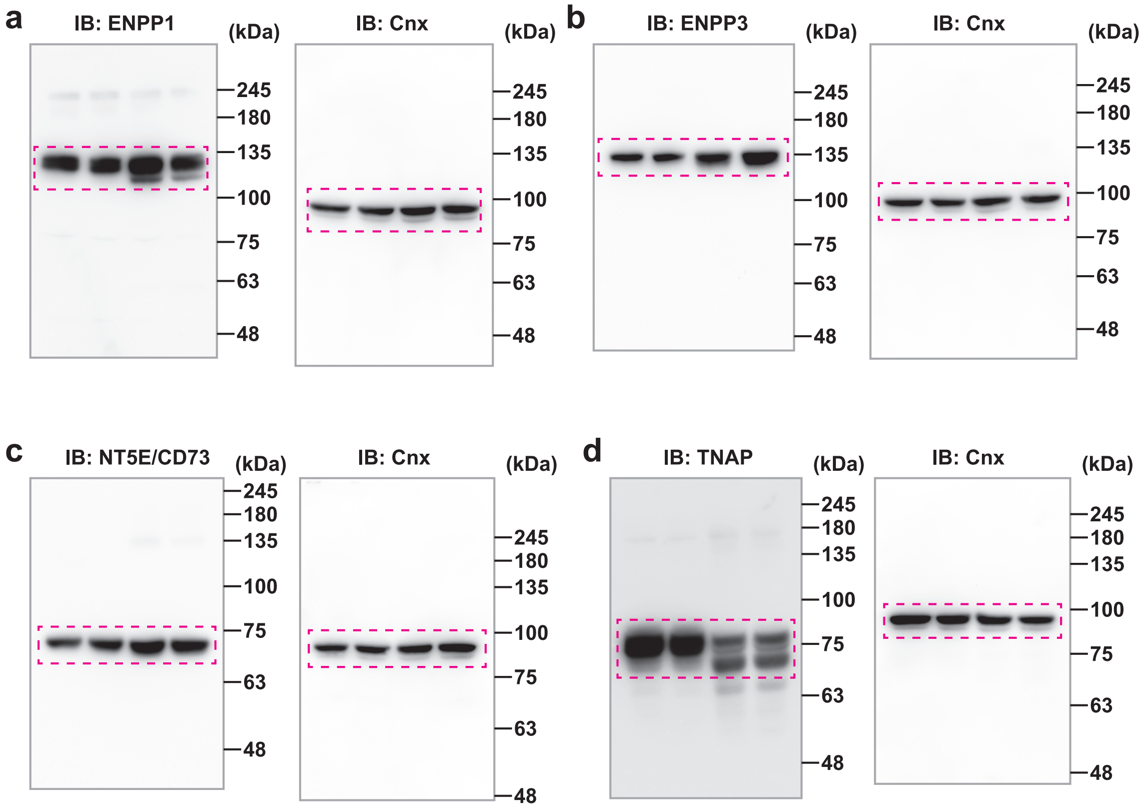

Supplementary Figure 9. Full-length immunoblot images used in Figure 7e-h. The panel used is boxed. The same blot was used sequentially (after stripping) for detection in each composite figure. The molecular weights of the marker proteins are indicated on the right of the immunoblot images.

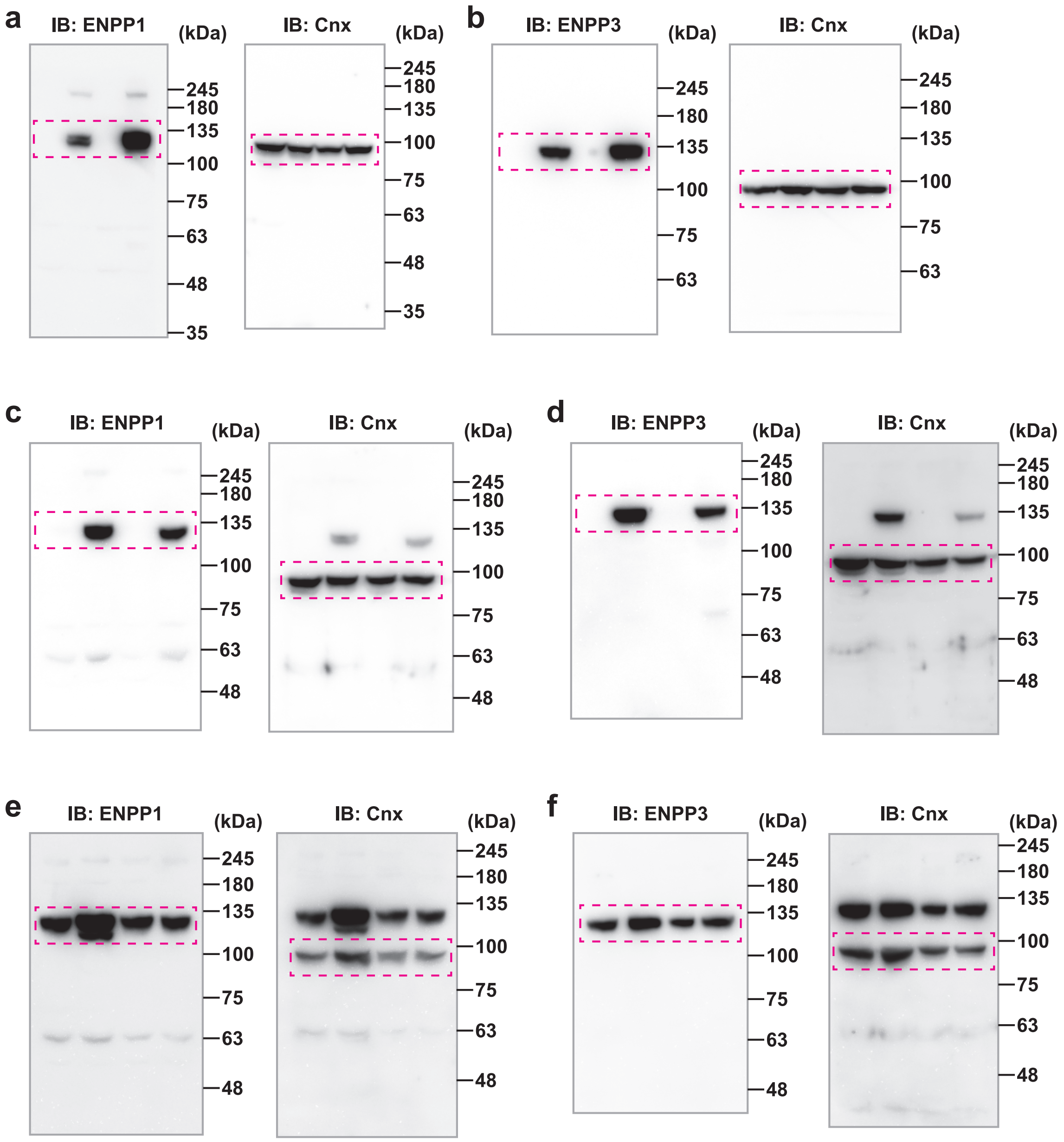

Supplementary Figure 10. Full-length immunoblot images used in Figure 8a-f. The panel used is boxed. The same blot was used sequentially (after stripping) for detection in each composite figure. The molecular weights of the marker proteins are indicated on the right of the immunoblot images.

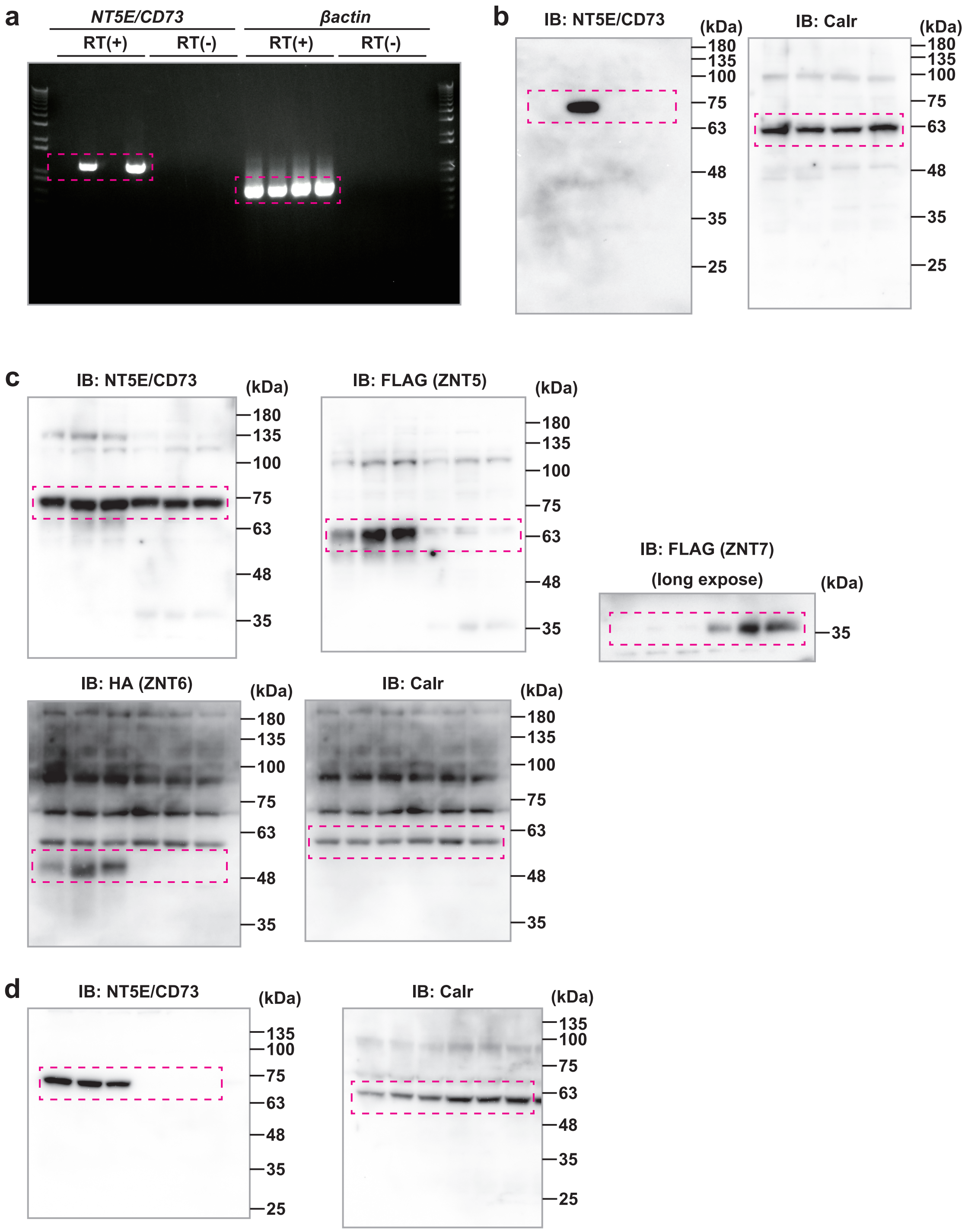

Supplementary Figure 11. Full-length gel and immunoblot images used in Figure 8g-k. The panel used is boxed. The same blot was used sequentially (after stripping) for detection in each composite figure. The molecular weights of the marker proteins are indicated on the right of the immunoblot images.

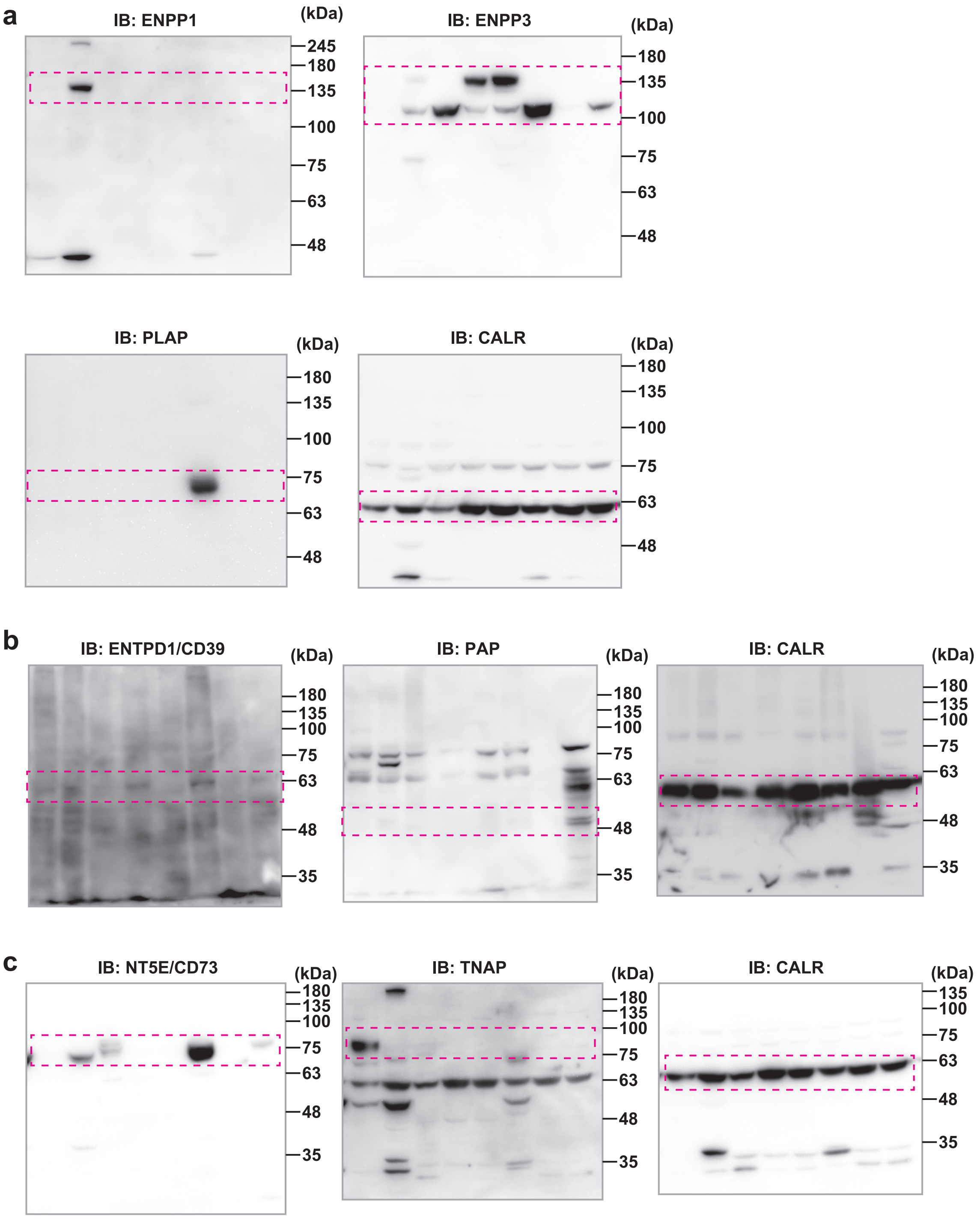

Supplementary Figure 12. Full-length immunoblot images used in Supplementary Figure 1. The panel used is boxed. The same blot was used sequentially (after stripping) for detection in each composite figure. The molecular weights of the marker proteins are indicated on the right of the immunoblot images.

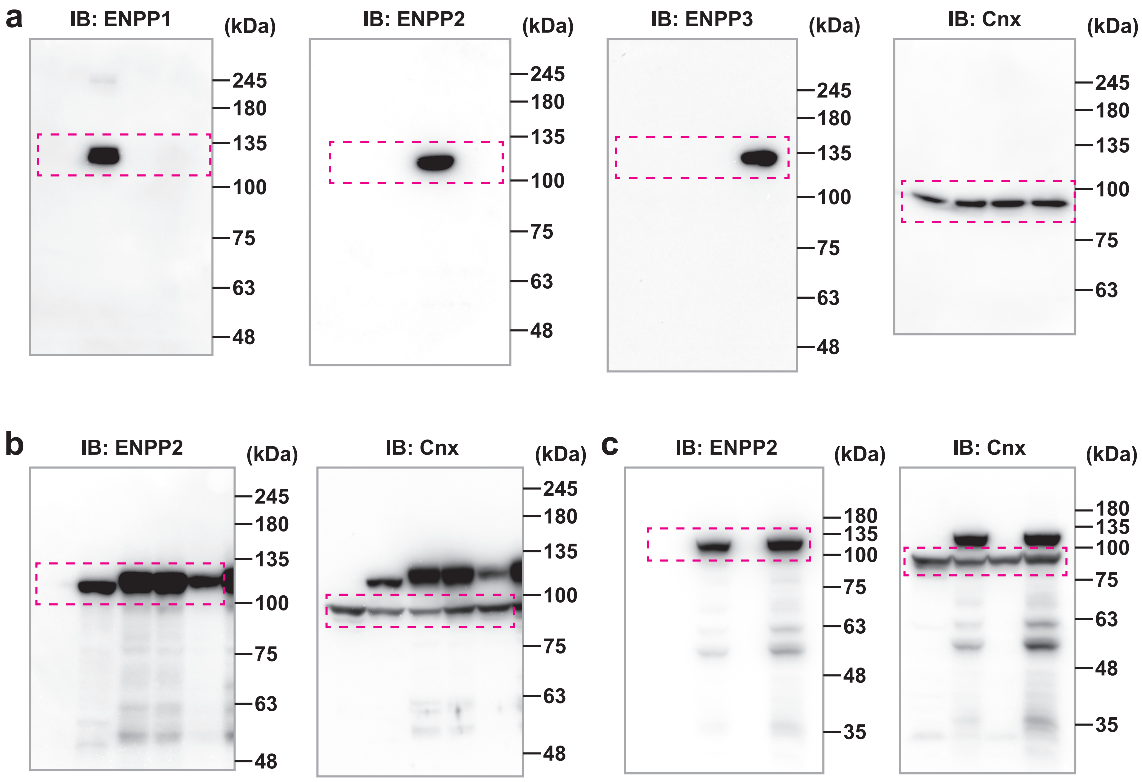

Supplementary Figure 13. Full-length immunoblot images used in Supplementary Figure 4. The panel used is boxed. The same blot was used sequentially (after stripping) for detection in each composite figure. The molecular weights of the marker proteins are indicated on the right of the immunoblot images.

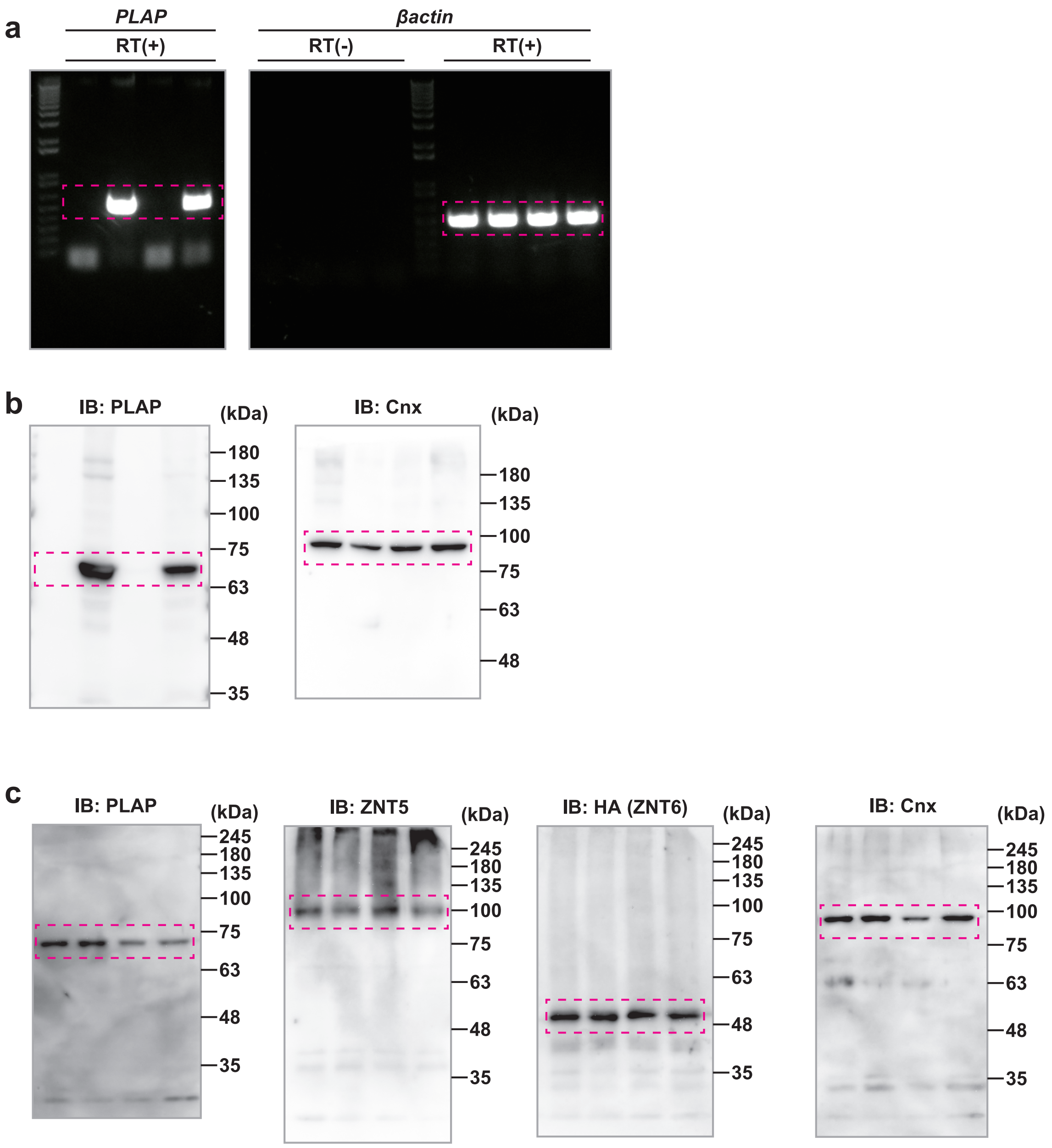

Supplementary Figure 14. Full-length gel and immunoblot images used in Supplementary Figure 5. The panel used is boxed. The same blot was used sequentially (after stripping) for detection in each composite figure. The molecular weights of the marker proteins are indicated on the right of the immunoblot images.

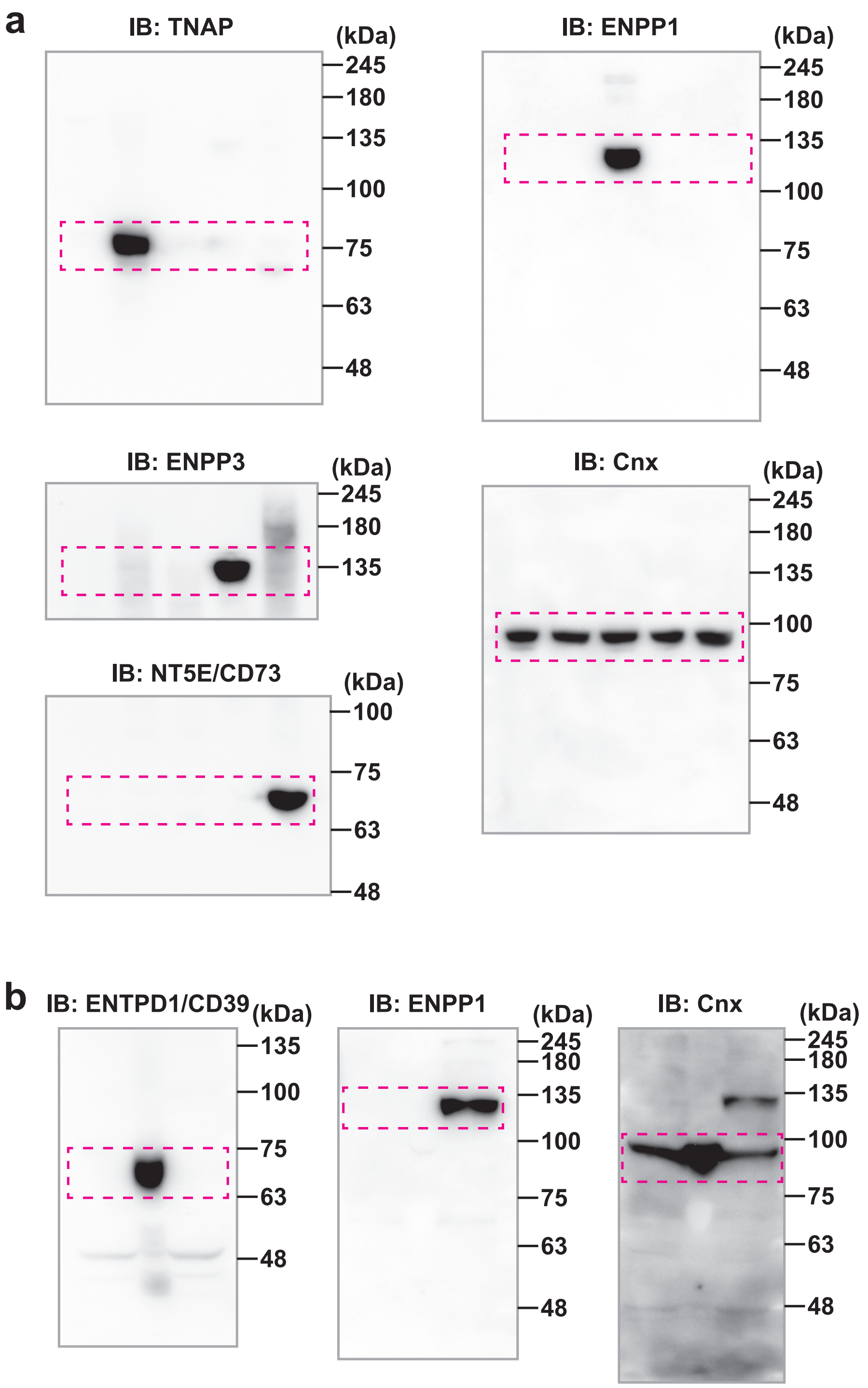

Supplementary Figure 15. Full-length immunoblot images used in Supplementary Figure 6. The panel used is boxed. The same blot was used sequentially (after stripping) for detection in each composite figure. The molecular weights of the marker proteins are indicated on the right of the immunoblot images.

Supplementary Table 1. Primer sequences used for PCR in this study.

Name	Sequence
NT5E/CD73-649F	5'-ACTCTAAATGTGAACAAAATTATTGCACTG-3'
NT5E/CD73-1845R	5'-CCTTCAGCTTCTAGGAGCCTGTAAAGATGG-3'
c*βactin*-431F	5'-GATCATGTTTGAGACCTTCAACACCCCAGC-3'
c*βactin*-1143R	5'-TGCTGATCCACATCTGCTGGAAGGTGGACA-3'
PLAP-318F	5'TGTAGACAAACATGTGCCAGACAGTGGAGC-3'
PLAP-909R	5'-GTCTCGGTGGATCTCGTATTTCATGTCTCC-3'

Supplementary References

1. Tsuji T*, et al.* Dissecting the process of activation of cancer-promoting zinc-requiring ectoenzymes by zinc metalation mediated by ZNT transporters. *J Biol Chem* **292**, 2159-2173 (2017).

2. Kato K*, et al.* Crystal structure of Enpp1, an extracellular glycoprotein involved in bone mineralization and insulin signaling. *Proc Natl Acad Sci U S A* **109**, 16876-16881 (2012).
